# Exploring Structural Insights of Aβ42 and α-Synuclein Monomers and Heterodimer: A Comparative Study Using Implicit and Explicit Solvent Simulations

**DOI:** 10.1021/acs.jpcb.4c00503

**Published:** 2024-05-03

**Authors:** Yuliia Varenyk, Panagiotis E. Theodorakis, Dinh Q. H. Pham, Mai Suan Li, Paweł Krupa

**Affiliations:** † Institute of Physics Polish Academy of Sciences, Al. Lotnikow 32/46, 02-668 Warsaw, Poland; ‡ Department of Theoretical Chemistry, University of Vienna, Vienna 1090, Austria

## Abstract

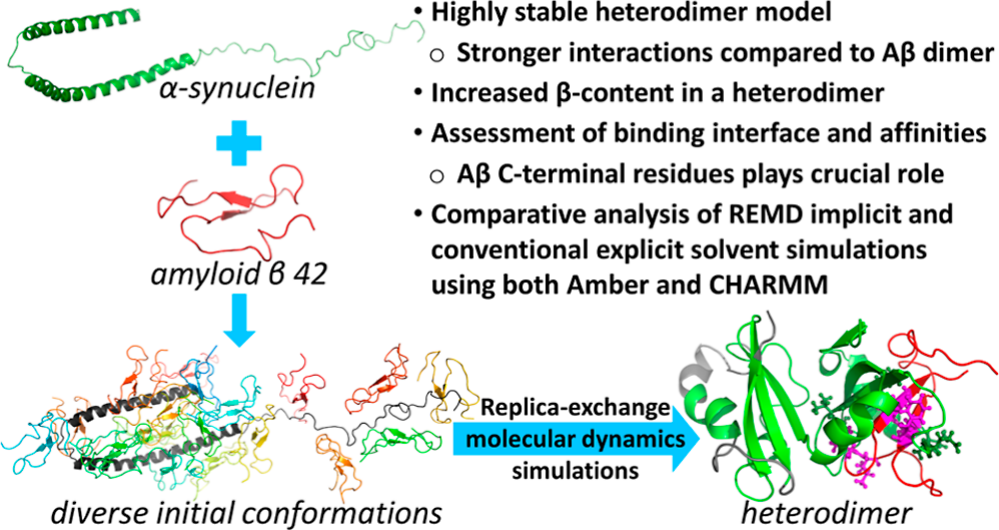

Protein misfolding, aggregation, and fibril formation play a central role in the development of severe neurological disorders, including Alzheimer’s and Parkinson’s diseases. The structural stability of mature fibrils in these diseases is of great importance, as organisms struggle to effectively eliminate amyloid plaques. To address this issue, it is crucial to investigate the early stages of fibril formation when monomers aggregate into small, toxic, and soluble oligomers. However, these structures are inherently disordered, making them challenging to study through experimental approaches. Recently, it has been shown experimentally that amyloid-β 42 (Aβ42) and α-synuclein (α-Syn) can coassemble. This has motivated us to investigate the interaction between their monomers as a first step toward exploring the possibility of forming heterodimeric complexes. In particular, our study involves the utilization of various Amber and CHARMM force-fields, employing both implicit and explicit solvent models in replica exchange and conventional simulation modes. This comprehensive approach allowed us to assess the strengths and weaknesses of these solvent models and force fields in comparison to experimental and theoretical findings, ensuring the highest level of robustness. Our investigations revealed that Aβ42 and α-Syn monomers can indeed form stable heterodimers, and the resulting heterodimeric model exhibits stronger interactions compared to the Aβ42 dimer. The binding of α-Syn to Aβ42 reduces the propensity of Aβ42 to adopt fibril-prone conformations and induces significant changes in its conformational properties. Notably, in AMBER-FB15 and CHARMM36m force fields with the use of explicit solvent, the presence of Aβ42 significantly increases the β-content of α-Syn, consistent with the experiments showing that Aβ42 triggers α-Syn aggregation. Our analysis clearly shows that although the use of implicit solvent resulted in too large compactness of monomeric α-Syn, structural properties of monomeric Aβ42 and the heterodimer were preserved in explicit-solvent simulations. We anticipate that our study sheds light on the interaction between α-Syn and Aβ42 proteins, thus providing the atom-level model required to assess the initial stage of aggregation mechanisms related to Alzheimer’s and Parkinson’s diseases.

## 1 Introduction

Amyloid-β (Aβ) and α-synuclein (α-Syn) are typically soluble proteins that can form neurotoxic aggregates associated with Alzheimer’s (AD) and Parkinson’s disease (PD), respectively. However, an overlap of pathologies is found in the case of dementia with Lewy bodies (DLB),^1−3^ which is a more general term to refer to diffuse DLB,^4^ DLB,^5^ cortical DLB,^6^ and senile DLB.^7^ Several studies suggest that Aβ and α-Syn coassemble, but the role of α-Syn in amyloid plaque formation requires further investigation.^2,8−12^ Here, we explore the possibility of forming heterodimeric structures from Aβ42 and α-Syn by means of all-atom molecular dynamics (MD) simulations. Toward our goal, we analyze various properties elucidating the behavior of these molecules in the heterodimer and the respective monomeric structures. Thus, we anticipate that our results might have implications in assessing aggregation mechanisms related to AD and PD.

α-Syn is a protein composed of an N-terminal domain (residues 1–60), a variable internal hydrophobic nonamyloid component (NAC) domain (residues 61–95), and a variable C-terminal acidic tail (residues 96–140), mainly consisting of negatively charged glutamate and aspartate residues.^11,13^ The middle 35-amino acid NAC domain is the building block of α-Syn aggregates.^14^ The structure of α-Syn depends on its environment, being natively unfolded in an aqueous solution, an α-helical conformation when bound to lipid vesicles, or β-pleated sheets in aggregates.^11^ In cells, two forms are found: an α-helix-rich membrane-bound form and a disordered free cytosolic form.^15^ Recent studies show that not only fibrils but also α-Syn and Aβ oligomers possess neurotoxic properties.^16−18^

Aβ has three positively and six negatively charged amino acids with its structure found in both membrane-associated and aqueous environments.^19,20^ Moreover, Aβ42 has a higher propensity for aggregation than Aβ40, despite representing only about 10% of the secreted Aβ,^21^ while it can act as a seed for Aβ40 aggregation in vitro.^22,23^ Given the importance of Aβ42 in the formation of Aβ plaques and the fact that α-Syn interacts differently with Aβ42 than with Aβ40,^24−27^ our focus in this study will only be on the interaction between Aβ42 and α-Syn. Previous in vitro and in vivo experiments have suggested a direct interaction between Aβ and α-Syn,^8,9^ confirmed in both human and mice brain samples with overlapping pathologies.^10,28^ Indeed, results obtained by NMR spectroscopy suggest that membrane-bound α-Syn interacts with membrane-associated Aβ42, while Aβ42 may also cleave the NAC fragment of α-Syn.^11^ Moreover, Aβ42 is eventually precipitated with the NAC according to neuropathological observations in DLB patients.^29,30^ In a recent experimental study by Köppen et al.,^31^ the effect of a minor concentration of Aβ42 and pGlu-Aβ42(3–42) on the aggregation propensity of α-Syn has been investigated. The authors found that the interaction of α-Syn with Aβ leads to accelerated fibril formation and enhanced nucleus formation. These effects can be explained to a certain extent by monitoring the structural changes of the two molecules upon their interaction. To this end, previous experimental studies have investigated the different structural forms of α-Syn and the different effects on Aβ aggregation.^32^ In particular, it has been found that fibrillar α-Syn favors the heterogeneous nucleation of Aβ aggregates, in contrast to monomeric α-Syn. The authors have attributed this effect to differences in concentration between the monomeric and the fibrillar α-Syn cases. Although these experiments have provided important insights into the Aβ and α-Syn coaggregation mechanisms, a detailed understanding of the interaction between these two molecules is still lacking. The development of structure prediction tools has drastically improved with the release of AlphaFold2,^33^ however, it also has its limitations, being unable to predict dynamics, the effect of the environment on structure and dynamics, and its efficiency drops down significantly for proteins for which there are no or very few structures available with similar sequences. For example, an attempt to use the AlphaFold-Multimer^34,35^ for the prediction of α-Syn–Aβ42 heterodimer results in a structure with a very low confidence score.

There are many approaches to lower the cost of computational studies and allow the study of larger systems over longer time scales, enabling the observation of large conformational changes in the studied systems rather than local fluctuations. For example, replica exchange MD^36,37^ simulations involve running many trajectories simultaneously at various temperatures, allowing for efficient crossing of energy barriers in higher temperature replicas. On the other hand, the use of graphical processing units (GPUs) can speed up simulation by approximately 2 orders of magnitude compared to the use of central processing units (CPUs).^38,39^ Although the use of GPUs is not limited to conventional MD simulations, its efficiency for REMD simulations is significantly lower.^40^ Another approach is to use simplified models, namely, coarse-grained models, in which the number of interaction centers and degrees of freedom is severely reduced, smoothing out the energy landscape. This allows for more than a 2–3 orders-of-magnitude speed-up.^41^ However, such methods can lose important details and are prone to inaccuracies, especially in the case of disordered systems. Another approach is to use an all-atom representation of the solute but use a mathematical function to describe its interaction with the solvent, called an implicit solvent model.^42^ This approach is especially useful if the studied system is largely disordered and forms noncompact conformations, as such systems would require large simulation boxes if explicit solvent would be selected, even in the presence of periodic boundary conditions. However, similarly to the use of a coarse-grained approach, the simplifications used may lead to inaccuracies.^43^

Although improvements in both hardware and software have allowed for the achievement of time scales of multiple microseconds in all-atom force fields for small and medium-size systems using GPUs, this is still far from the fibril-formation time scales of hours and days. Therefore, in this work, we focused on the initial aggregation events related to monomeric Aβ42 and α-Syn, as well as their heterodimer, in implicit and explicit solvent simulations instead. Due to computational restrictions, large-scale implicit solvent REMD simulations in the Amber all-atom force field were coupled with GPU-accelerated conventional MD explicit solvent simulations in AMBER-FB15^44,45^ and CHARMM36m^46^ force fields. Based on a detailed atomic-scale analysis of a range of properties, we identified key conformational changes for α-Syn and Aβ42 monomers, as well as interactions that crucially contribute to different behaviors in the heteromeric structure compared to the respective monomeric structures. Moreover, we confirmed the possibility of heterodimer formation, identified its structure, and provided a comparison between implicit and explicit solvent treatments of monomeric and heterodimeric forms of both molecules, including multiple Amber and CHARMM36m force fields. Thus, we anticipate that our results will have further implications for a better understanding of the coassembly of α-Syn and Aβ42 chains.

## 2 Methods

### 2.1 Initial Structures Used in the Simulations

**Figure 1:**
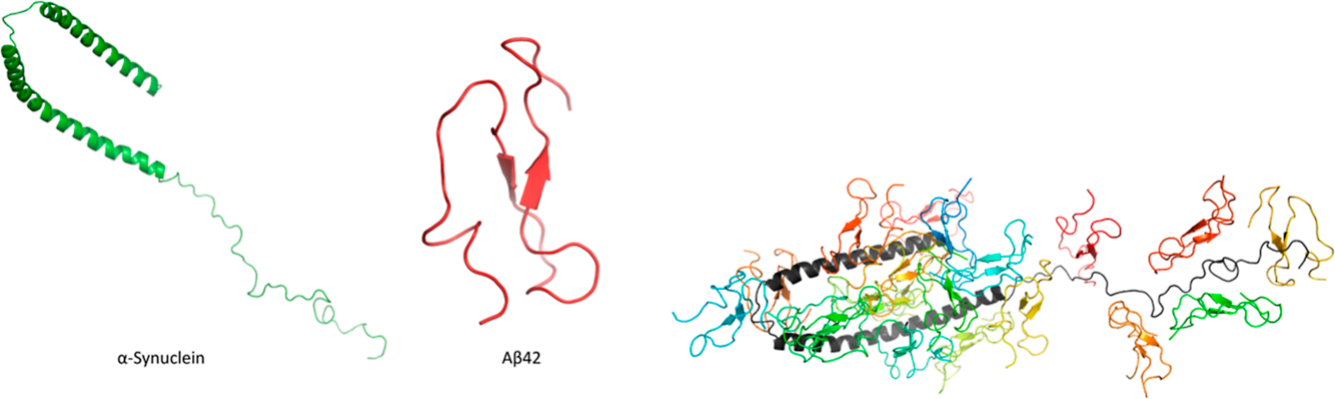
Cartoon representations of monomeric α-Syn (PDB id: 1XQ8), Aβ42,^47^ and 20 orientations of α-Syn–Aβ42 heterodimers used as initial configurations in the REMD simulations.

In this study, we employed MD to simulate three different systems: monomeric α-Syn, monomeric Aβ42, and their heterodimer (Figure 1). The initial structure of α-Syn was adapted from the Protein Data Bank (PDB id: 1XQ8), while the initial structure of Aβ42 is the one proposed by Yang and Teplow^47^ (Figure 1). MD simulations of monomeric systems were utilized to evaluate and compare the performance of implicit and explicit solvent approaches, both with each other and with experimental data. Additionally, they were used to estimate conformational changes upon heterodimer formation.

We generated 20 initial conformations of the heterodimer using the preparatory step of the UNRES-Dock algorithm.^48^ This step places chains of the molecules in as different orientations as possible, with the condition that the chains have to form at least one interaction. Using such an approach, instead of complete docking, allowed us to avoid any bias arising from the initial structures being stuck in the local minimum of the energy and speed up the equilibration process. Moreover, it was not expected for the monomeric forms of Aβ42 and α-Syn to remain in the initial conformations during REMD simulations due to their intrinsically disordered character and the fact that the monomeric α-Syn conformation was obtained experimentally in a micelle, and it substantially varies from the aqueous environment.

**Figure 2:**
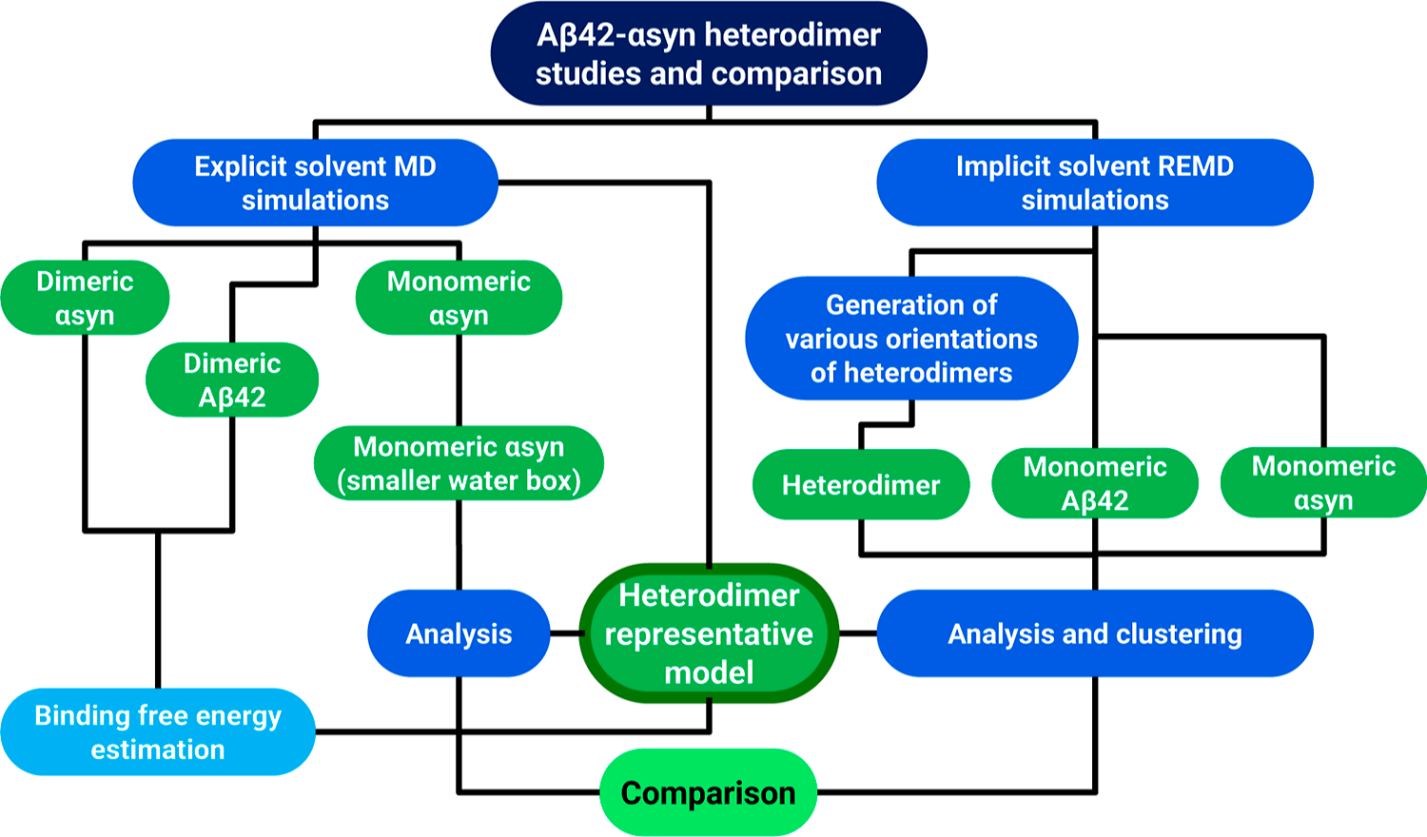
Schematic visualization of the flowchart presenting simulations and analyses performed in this work.

The types of MD simulations performed in this study are summarized in Table 1, while additional information can be found in Table S1. The workflow of the simulations is shown in Figure 2. Initial and final conformations from each simulation are attached to the Supporting Information.

**Table 1:** Types of Simulations Performed in This Study: REMD Simulations in Implicit Solvent of Aβ42 and α-Syn Monomers and Their Heterodimer; Folding Conventional MD Simulations of α-Syn in Explicit Solvent; Stability Determination of Aβ42-α-Syn Heterodimer in Explicit Solvent Conventional MD Simulations; Conventional MD Simulations to Estimate Binding Affinity of Aβ42 and α-Syn Dimers

system	force field	solvent	trajectories	total time [μs]	temperature [K]
Aβ42	Amber ff14SBonlysc	GB-Neck2	20 × 4000 ns	80	281–512.19
α-Syn	Amber ff14SBonlysc	GB-Neck2	20 × 2000 ns	40	281–512.19
Aβ42-α-Syn	Amber ff14SBonlysc	GB-Neck2	20 × 1150 ns	23	281–512.19
α-Syn	AMBER-FB15	TIP3P-FB	3 × 1667 ns	5	300
α-Syn	CHARMM36m	TIP3P*	3 × 1667 ns	5	300
Aβ42-α-Syn	AMBER-FB15	TIP3P-FB	5 × 1000 ns	5	300
Aβ42-α-Syn	CHARMM36m	TIP3P*	5 × 1000 ns	5	300
α-Syn-α-Syn	AMBER-FB15	TIP3P-FB	3 × 5 × 50 ns	0.75	300
Aβ42-Aβ42	AMBER-FB15	TIP3P-FB	3 × 5 × 50 ns	0.75	300

### 2.2 Implicit Solvent Simulations of Monomers and the Heterodimer

To extensively search the conformational space of monomeric and heterodimeric forms of α-Syn and Aβ42, the replica exchange molecular dynamics (REMD)^36,37^ sampling method was selected, as it allows crossing energetic barriers at higher temperatures. However, this approach requires the use of many trajectories (replicas) to ensure proper performance and cannot be efficiently performed with GPU acceleration. Moreover, due to the intrinsically disordered character of both α-Syn and Aβ42, there is an abundance of extended molecular conformations. Hence, a large simulation box is required, especially in the case of α-Syn (with a maximum distance between atoms in PDB 1XQ8 close to 160 Å), to avoid the interaction between periodic images of the molecule.

This translates to large requirements for excessive computation time when explicit solvent conditions are considered, and GPU-accelerated simulations are unavailable for extensive REMD sampling. Therefore, we used an implicit solvent model to conduct our simulations, resulting in a significant speed-up compared to the corresponding explicit solvent model, which should not compromise the accuracy of the simulations.^45,49^ In particular, the implicit generalized Born scheme was used to simulate our systems, as implemented in the AMBER20 package,^50^ using the ff14SBonlysc force-field with mbondi3 radii.^49^ Several studies suggest that the combination of ff14SBonlysc with the GB-Neck2 model provides reasonable results regarding the prediction of protein folding mechanisms.^45,49^ Moreover, the standard version of AMBER ff14SB was found to provide good agreement with available experimental data for Aβ.^51,52^

To deal with the rough energy landscape that stems from the use of all-atom force-fields and enhance the sampling of the conformational space, we carried out REMD simulations with 20 replicas with temperatures ranging from 281 to about 512 K (Table S2). The temperature was controlled through a Langevin thermostat for each replica, and the equations of motion were integrated with a leapfrog algorithm as implemented in the AMBER20 package.^50^ The temperature distribution of the replicas was generated by a temperature generator^53^ to provide satisfactory exchange rates.

To prepare monomeric and heterodimeric systems for the REMD simulations, their energy was minimized using 7000 steps of the steepest descent and 3000 steps of the conjugate gradient method. Then, the systems were gradually heated up and relaxed at each individual temperature for 5 ns by employing conventional simulation in the canonical ensemble with the Langevin thermostat. Simulations using the implicit solvent model were performed with an infinite cutoff for long-range interactions. During the REMD simulations, exchanges between the different replicas were attempted every 500 steps, where the time step in the simulations was set to 2 fs.

Trajectories for monomeric systems reached a length of 4 μs for Aβ42 and 2 μs for α-Syn per replica, translating into 80 and 40 μs of total simulation time, respectively, while the heterodimeric system was simulated for 1 μs (20 μs of total simulation time) due to its larger size.

To ensure proper sampling in the case of the heteromeric system, each replica started from a different initial orientation of the chains (Figure 1), as mentioned in the above section. The analysis of our results is based on the trajectories obtained from the REMD simulations for both the monomeric and the heteromeric systems at temperature *T* = 300.69 K. It should be noted that this temperature is lower than the physiological temperature of approximately 310 K. However, it is a typical temperature at which protein force fields are usually optimized and tested and at which most simulations are performed. Therefore, it was selected to ensure high reliability and consistency with existing data, however, it is expected that there should not be any substantial changes between trajectories at 300 and 310 K.

### 2.3 Explicit Solvent Simulations of Monomers and the Heterodimer

To compare the behavior of the studied systems between implicit and explicit solvent models and to ensure the highest level of robustness, an additional series of explicit solvent simulations for monomeric α-Syn and the heterodimer were run in two completely different force fields. As we had previously simulated monomeric Aβ42 using multiple force fields and two sampling methods (REMD and conventional MD) in a multiple-microsecond time scale,^51^ we decided to use this data for comparison. Similar to implicit solvent simulations, a Langevin thermostat was used to maintain a temperature of 300 K, while a cutoff of 9 Å was applied for long-range interactions with the PME method.^54^ The selection of the AMBER-FB15 force field was dedicated to its ability to better represent conformational fluctuations of the studied systems away from equilibrium, while coupling with the TIP3P-FB water model provides higher and closer-to-experiment values of the radius of gyration,^55^ which is expected for disordered systems. CHARMM36m is a modification of the CHARMM36 force field,^56^ which should provide satisfactory accuracy for both folded and intrinsically disordered peptides and proteins^46^ and has been found to be among the top force fields to study various IDPs.^57^ Parameters for the AMBER-FB15 force field were generated using tLeap, part of the AmberTools package, while CHARMM36m parameters were generated by the use of the CHARMM-GUI server.^58^ In explicit solvent simulations, all protein systems were surrounded by a layer of water using the recommended water model, while Na^+^ and Cl^–^ ions were added to neutralize the charge and reach a physiological concentration of approximately 150 mM.

#### 2.3.1 Folding of the Monomeric α-Syn

The experimental monomeric α-Syn structure (PDB: 1XQ8) is in an extended conformation, as it was bound to a micelle in the NMR experiment, therefore, it is expected that its conformation in solution would be significantly different. Therefore, we first ran a short 100 ns run in a very large explicit solvent box (165 × 165 × 165 Å) with approximately 112,000 water molecules to allow the molecule to reach a more compact structure. Then, a second series of MD simulations (each consisting of three 1.67 μs trajectories, resulting in a total time of 5 μs per system) was run, as previously mentioned, but in a significantly smaller periodic box (91 × 91 × 91 Å) with approximately 19,000 water molecules both in AMBER-FB15 and CHARMM36m force fields.

#### 2.3.2 Studies of α-Syn–Aβ42 Heterodimer Stability

A series of five trajectories, each of 1 μs length, resulting in a total time of 5 μs per system, starting from the most probable conformation of the α-Syn–Aβ42 heterodimer, was performed with the AMBER-FB15 force field^55^ and the TIP3P-FB water model,^59^ and the CHARMM36m force field with the modified TIP3P model. The use of a different force field compared to the implicit solvent simulations was motivated by the need to verify the stability of the heterodimer regardless of the simulation details.

The system was neutralized and placed in a periodic boundary box together with approximately 20,000 water molecules, providing a layer of about 18 Å around the protein complex. Due to the use of the equilibrated structure for the heterodimer, a reasonable size of the periodic box could be employed, in contrast to the implicit solvent simulations where extended disordered conformations were used as initial configurations for the simulations. The system’s energy was minimized, followed by equilibration (1 ns), and then production runs, consisting of five trajectories, each of 1 μs, were performed in explicit solvent.

#### 2.3.3 α-Syn–Aβ42 Heterodimer and Aβ42 Dimer Binding Energies

To compare the binding free energies, five short MD simulations (each of 50 ns) were conducted for three Aβ42 models and three α-Syn dimeric models, as predicted in previous studies.^60,61^ The simulations used the same conditions as the explicit water simulations described above, employing the AMBER-FB15 force field^55^ and the TIP3P-FB water model.^59^ The last 10 ns of the simulations were used to perform the MM-PBSA analysis, providing an estimate of the binding free energy for each system. Predicted values were subsequently averaged over five trajectories for each system.

### 2.4 Analysis

#### 2.4.1 Root Mean Square Deviation

The first property we have considered in our study is the root mean square deviation (rmsd) of the atomic positions of the molecules, which is a first step toward validating the stability of our simulations. The rmsd is defined as follows
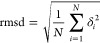
1where δ_*i*_^2^ is the distance between atom *i* and its position at the reference structure, which contains *N* atoms. As usual, only the heavy atoms (e.g., N, C, O, etc.) are considered in the rmsd calculation and the usual alignment procedure takes place before applying the above equation.

#### 2.4.2 Radius of Gyration

As a next step in our analysis, we have monitored the radius of gyration (*R*_g_) and the maximum radius of gyration (*R*_g max_) for each chain in both the monomeric and the heteromeric cases. *R*_g_ is described by the following relation
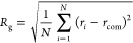
2where *r*_com_ is the center of mass of the chain. *N* is the number of heavy atoms in each chain. *R*_g max_ is the distance of the most distant atom from the center of mass.

#### 2.4.3 Other Analysis

For analyzing properties other than rmsd and *R*_g_, we utilized the second halves of the simulation trajectories, considering them as equilibrated portions. Secondary structure elements were determined using the DSSP algorithm^62^ as implemented in the CPPTRAJ tool.^50^ Solvent-accessible surface area (SASA) concerning individual residues and the whole molecules was calculated using the Linear Combinations of terms composed from Pairwise Overlaps (LCPO) method.^63^ Additionally, we calculated contact maps for both monomeric and heteromeric cases. A contact was considered when at least two heavy atoms from different amino acid residues were within a distance of 0.5 nm, under the condition that in a single snapshot, only one contact could be formed within a single pair of residues. We employed a hierarchical agglomerative average-linkage clustering method to divide the ensemble of structures into five groups, and then the cluster centroid of the largest cluster was treated as the most representative structure. Free energy maps were calculated as described in our previous work (eq 6 and its description in ref (64)).

#### 2.4.4 Binding Free Energy Analysis

Binding free energies were estimated using two approaches. In the first one, a subset of snapshots from MD trajectories was analyzed by means of the MM-PBSA method as implemented in AMBER software.^50^ Due to performance limitations, 100 snapshots were used to calculate enthalpic contributions, while 10 snapshots were used to estimate entropic contributions using the normal-mode analysis method, which is a standard procedure to predict binding affinities from MD trajectories.^65^ In the second approach, single representative models were used instead of ensembles of structures to predict global and per-residue binding free energies using the HawkDock server,^66^ which we found to provide reasonable relative values.^67^

#### 2.4.5 Molecular Mechanics—Poisson–Boltzmann Surface Area Method

By using the MM-PBSA method, the binding affinity (binding free energy) (Δ*G*_bind_) between two molecules is composed of the following five terms
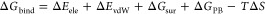
3where Δ*E*_ele_ and Δ*E*_vdW_ are, correspondingly, electrostatic and van der Waals interaction energy; Δ*G*_sur_ and Δ*G*_PB_ are nonpolar and polar solvation energies; and *T*Δ*S* is the entropic contribution.

MMPBSA.py, a part of AmberTools, was used to calculate the binding free energy. Charges and atomic radii from the force field used for the simulation were used for calculating electrostatic and van der Waals interactions. The polar solvation energy was calculated by solving the Poisson–Boltzmann equation using the PBSA program, also a part of AmberTools, at an ionic concentration of 0.15 M. The internal and external dielectric constants were set to 1.0 and 80.0. The nonpolar solvation energy is proportional to the SASA as follows
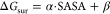
4where α and β were set to 0.005 and 0.0, respectively. SASA was calculated by the LCPO method^63^ with a solvent probing radius of 1.4 Å. A normal mode approximation calculation was used to obtain the entropic contribution term at a temperature of *T* = 300 K.

## 3 Results and Discussion

### 3.1 Implicit Solvent Simulations of Monomeric α-Syn and Aβ42 and Their Heterodimer

The first step of the analysis involved checking whether the REMD simulations were properly configured to ensure satisfactory exchange rates and temperature walks. It is known that problems with achieving a proper exchange rate, without obtaining a good walk between various temperature replicas, in which only local exchanges between 2 and 3 neighboring replicas are observed, could effectively decrease the sampling efficiency of the REMD simulation. This effect usually worsens with an increase in system size, including both solute and solvent, and in explicit solvent simulations, it can even be observed for small systems such as monomeric Aβ42.^51^ However, analysis of the implicit solvent trajectories generated in this study clearly shows that most of the trajectories successfully traverse wide ranges of temperatures (Figure S1). Even those limited to narrower temperature ranges travel between at least 7 different temperature replicas (e.g., in the temperature range of approximately 281–343 K), allowing for proper conformational search of the system. This indicates that the REMD simulations were well-configured and should provide efficient sampling of the conformational space for the studied systems.

#### 3.1.1 Analysis of Structure Stability and Shape

We analyzed the time evolution of the rmsd (Figure S2) from the initial structures (Figure 1), revealing high stability of all systems throughout the simulations. Notably, α-Syn, in both monomeric and heterodimeric configurations, exhibited an initial hydrophobic collapse (Figure 1). This observation aligns with expectations, given that the initial experimental structure was derived after binding to the micelle and simulations were conducted in a solution environment.

**Figure 3:**
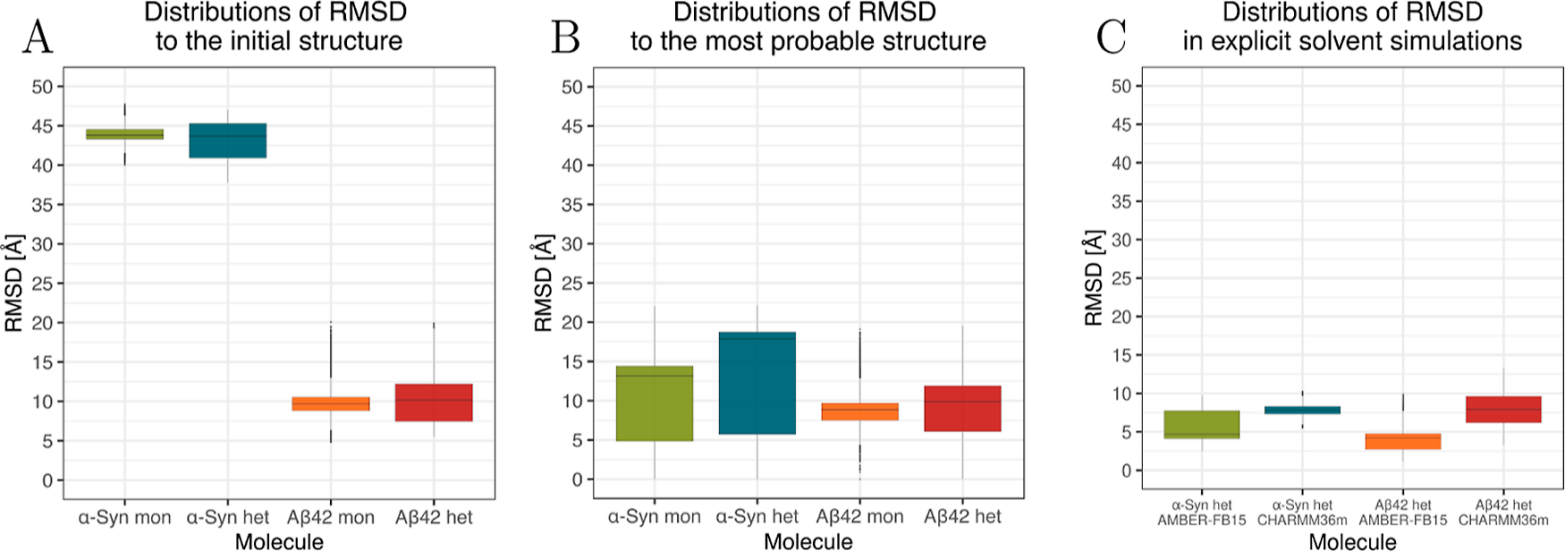
rmsd distributions of α-Syn and Aβ42 as monomers (mon) and in the heterodimer (het), as indicated. (A) initial, and (B) the most probable minimum free energy structure used as a reference for rmsd calculations.

In particular, the rmsd values of α-Syn are approximately four times higher than those of Aβ42, a result of an extended initial structure and a larger chain length (Figure 3). The equilibrium rmsd values for both systems are generally high due to the disordered character of the molecules. By comparing the differences between the monomeric α-Syn and Aβ42 systems with the heteromeric one, we observe that the average values are similar. However, both α-Syn and Aβ42 molecules show larger standard deviations in the rmsd values in the heteromeric system than in the respective monomeric systems. Additionally, this difference is more pronounced in the case of Aβ42. This may be attributed to the interaction between α-Syn and Aβ42 in different orientations for various replicas, leading to average values with a larger variance. However, this observation is expected for systems that are highly disordered, especially in monomeric forms.

**Figure 4:**
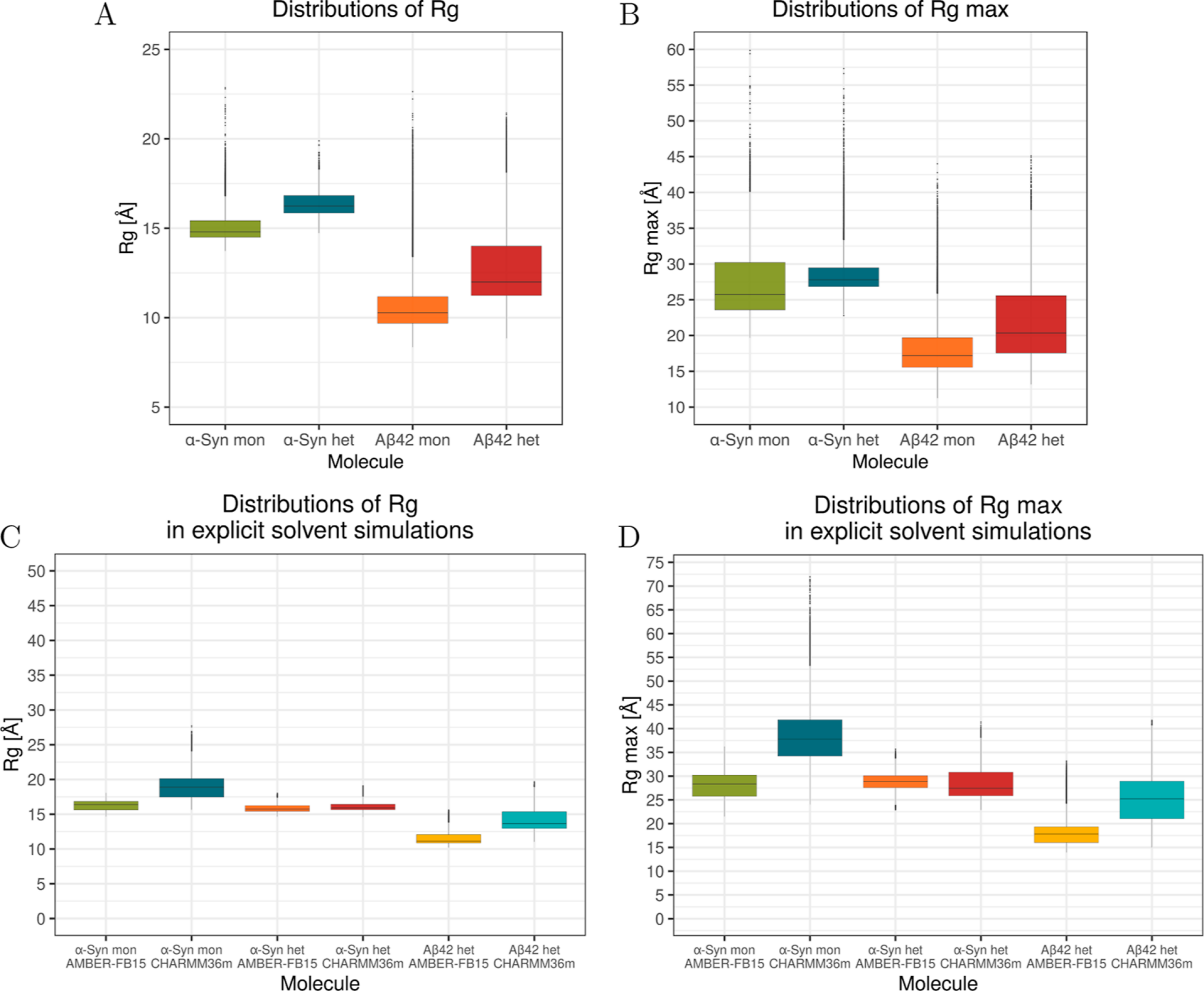
*R*_g_ (A) and *R*_g max_ (B) distributions of α-Syn and Aβ42 as monomers (mon) and in the heterodimer (het) as indicated.

Our findings reveal that both α-Syn and Aβ42 exhibit larger dimensions in heteromeric systems compared to their respective monomeric counterparts (Figure 4). Although the increase in *R*_g_ is subtle, it is more noticeable for Aβ42. However, this trend is not as pronounced when examining *R*_g max_, as the most distant residues from the center of mass occupy comparable positions in monomeric and heteromeric systems, despite the more compact conformation of α-Syn. Generally, *R*_g_ values around 15 Å for α-Syn suggest a predominantly compact conformation during REMD simulations.^68,69^ Our results imply that α-Syn may become more stable upon binding to Aβ42. Additionally, the larger standard deviation in *R*_g_ and *R*_g max_ between monomeric and heterodimeric forms is more pronounced for Aβ42. In summary, the analysis of rmsd, *R*_g_, and *R*_g max_ collectively suggests that the Aβ42 chain is more influenced by the presence of α-Syn than vice versa.

Experimental *R*_g_ of Aβ42 is approximately 10.1–10.6 Å,^70,71^ which closely aligns with the average *R*_g_ observed in our implicit solvent simulations of monomeric Aβ42. These results are also similar to the values observed in explicit solvent simulations.^51^ On the contrary, the NMR-calculated hydration radius of α-Syn is about 29 Å. Considering the nonspherical shape of monomeric α-Syn, this would indicate an *R*_g_ above 23 Å, while the analysis of data from SAXS suggests an even larger value of 35.5 Å.^72^ These values are considerably larger than the *R*_g_ range observed in our implicit solvent simulations, which focuses on the range of 14–16 Å. Analysis of the explicit solvent simulations provided an average *R*_g_ of 16.20 Å (SD: 0.54 Å) and 18.55 Å (SD: 0.42 Å) for simulations with AMBER-FB15 and CHARMM36m force fields, respectively. Although the values in CHARMM36m were higher (18.55 Å, SD: 0.42 Å), they are still significantly lower than those observed in the experiments.^72^ However, it should be noted that experimental conditions may affect the monomeric α-Syn conformation, and it is challenging to ensure that α-Syn did not start to aggregate, leading to potential differences in these values. The average observed values in our simulations and other studies^72^ are notably lower than those experimental values.

#### 3.1.2 Free Energy Maps and Minimum Energy Structures

We have calculated the free energy maps as a function of *R*_g_ and rmsd for α-Syn and Aβ42 in the monomeric and the heteromeric cases (Figure S3). For both chains, the free energy maps for the heterodimer exhibit similar behavior, with multiple energy minima being distinct from each other in comparison with the more homogeneous free energy maps in the case of monomeric structures, where mostly a single minimum can be easily identified. In the case where the number of contacts is chosen as an order parameter instead of the rmsd, a single free energy minimum is mostly observed for all cases (Figure S4). By using cluster analysis, we have identified the most probable structure for α-Syn and Aβ42, and we have recalculated the rmsd with respect to these structures. Clearly, the rmsd is significantly smaller in the case of α-Syn (about 14 Å) and also smaller in the case of Aβ42 (about 9 Å, Figure 3). Both α-Syn and Aβ42 chains exhibit larger rmsd in the heteromeric case, where the standard deviation is smaller in the case of α-Syn than in the case of Aβ42. This shows that the α-Syn molecule exerts a greater influence on Aβ42 than Aβ42 on α-Syn.

#### 3.1.3 Fluctuations of Structure Elements during Simulations

By using average conformations from the respective trajectories as reference structures for α-Syn and Aβ42, we calculated the root-mean-square fluctuations (RMSF) for each residue (Figure S5). Interestingly, the differences between the monomeric and heteromeric cases are small for Aβ42. In contrast, the α-Syn atoms in the heterodimer case exhibit larger fluctuations from their average positions compared to those in the α-Syn monomeric case. In particular, significant differences occur along the entire α-Syn chain, especially in residues 26–63, 98–107, and 116–123. Experimental studies by Gallardo et al. show that although the N-terminus of α-Syn has some helical propensity, the C-terminus is disordered due to its acidic character,^73^ which is consistent with the high structural fluctuation of the C-terminus in the heterodimer observed in our study. Although rmsd suggests otherwise, the local fluctuations of α-Syn atoms (RMSF) show larger deviations from their reference positions than those seen in the case of Aβ42. However, it should be noted that RMSF analysis is performed with respect to the average conformations, which can significantly vary and provide only a rough estimation due to the disordered character of the studied molecules, especially in the case of a heterodimer, in which in addition to the conformations close to the most probable orientation of the molecules, a small number of snapshots with less probable orientations can be present due to the nature of REMD simulations, increasing the base value of the RMSF. Most of the less flexible fragments of α-Syn are located in the regions interacting with Aβ42, e.g., residues 24–26, 69–71, and 109–115.

#### 3.1.4 α-Syn–Aβ42 Heterodimer Allows for the Formation of the Hydrophobic Core between the Molecules

**Figure 5:**
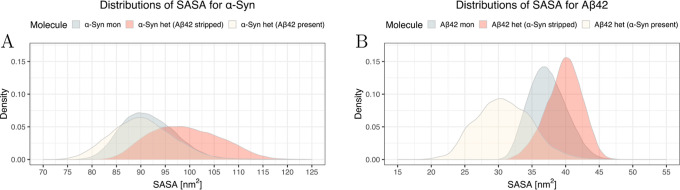
Distributions of SASA for (A) α-Syn and (B) Aβ42 in monomer (mon) and heterodimer (het) simulations, as indicated.

The comparison of SASA values in monomeric and heterodimeric forms (Figure 5) shows that there is no significant difference for α-Syn. This can be attributed to the size discrepancy between the molecules and the presence of a large unbound part of α-Syn to Aβ42, which compensates for the SASA difference. The presence of α-Syn is causing a drastic change to the Aβ42 SASA, which decreases by 7 nm^2^, namely, from 37 to 30 nm^2^. This significant difference suggests a higher hydrophobicity of Aβ42 compared to α-Syn. In general, SASA values for monomeric Aβ42 are in agreement with previous observations, where high flexibility of the chains was obtained,^74,75^ e.g., by using REMD sampling.^51^

There are substantial differences in SASA values for Aβ42 upon binding to α-Syn: the average SASA decreases by about 20%, however, when considering the theoretical SASA of Aβ42 in the heterodimer with α-Syn removed from analysis, the average SASA increases by about 11%. This suggests that interactions between complexes predominantly involve hydrophobic residues, forming a hydrophobic core upon complex formation to shield these residues from water molecules. The SASA difference per residue (Figure S6) affirms that binding is more favorable for Aβ42 than for α-Syn, as the former can effectively reduce unfavorable interactions with water. Notably, the difference in per residue SASA upon binding is most pronounced (above 0.4 nm^2^) for specific Aβ42 residues: Leu17, Phe19, Phe20, Ile31, and Ile32.

The total size of the binding interface of the α-Syn–Aβ42 heterodimer is equal to 10 nm^2^ for each of the molecules (this translates to approximately 11 and 33% of Aβ42 and α-Syn, respectively, being involved in the interactions within the heterodimer), indicating a significant reduction in hydrophobicity within the binding region. This also elucidates why the impact of α-Syn on Aβ42 is much larger than the impact of Aβ42 on α-Syn, as a larger portion of Aβ42 is involved in interactions with its partner in the heterodimer. It should be noted that the varying degrees of the effects of one system on the other are also caused by the size difference between the molecules, with α-Syn having 233% more amino acid residues than Aβ42. As the hydrophobic interactions between α-Syn and Aβ42 are important for the formation of the heterodimer and the presence of α-Syn causes a drastic change to the Aβ42 SASA, it indicates a higher hydrophobicity of Aβ42 compared to α-Syn. This, together with significant size difference, likely makes Aβ42 more susceptible to the influence of α-Syn binding compared to the reverse situation.

#### 3.1.5 Secondary Structure Content

In the absence of α-Syn, the β-content of Aβ42 (27.92%) (Table 1) is consistent with the result (26.2%) obtained by using the OPLS/AA force field and GB implicit solvent,^76^ but much higher than the 10% predicted by the AMBER force field ff99SB and water model TIP3P.^60^ Overall, our result falls into the experimental^26,77^ and theoretical^51,78−81^ range of 9–27%. Similar to previous works (see Review^82^), the helix structure (9.97%) (Table 1) is less populated than the β strand, and the coil is more abundant than the turn. Experimental circular dichroism (CD) spectroscopy studies estimate the α-helical content between 3 and 9% for monomeric Aβ42.^26,77^ In our simulations, the helix structure is slightly more pronounced, which is partially caused by the use of implicit solvent. Nevertheless, this value is in the range found in other MD studies.^51^

The analysis of secondary structure for both the α-Syn and the Aβ42 (Table 2) indicates that in the case of the heterodimer, the β-sheet content slightly increases, whereas the α-helix content decreases. The turns remain the same for Aβ42, while a decrease is observed in the case of α-Syn. Finally, the coil increases for both α-Syn and Aβ42 in the heterodimer case. All these changes are not statistically important due to the large standard deviations of secondary structure contents. Experimental studies suggest that monomeric α-Syn possesses a high α-content upon contact with lipids, which shifts to an abundance of β-structures in water.^83^ In our studies, α-Syn is present in the intermediate state, in which both α- and β-contents are high due to the disordered character of the molecule. In general, our results are in agreement with experimental and discrete MD (DMD) studies, which showed that α-Syn can form both α-helices (mostly residues 8–32) and β-sheets (mostly residues 35–56 and 61–95), while the C-terminus remains unstructured.^73,84^

**Table 2:** Average Secondary Structure Content Obtained in This Study for Monomeric Aβ42, α-Syn in Implicit and Explicit Solvent and Their Heterodimer and Literature Ranges for Monomeric Aβ42^a^

molecule	FF_solvent_	β-sheets (%)	α-helix (%)	turn (%)	coil (%)
Aβ42_mon_	ff14SBonlysc_GB__–__Neck2_	27.92 (7.18)	9.97 (4.64)	20.68 (3.24)	41.43 (4.57)
Aβ42_mon_^51^	CHARMM36m_TIP3P*_	23.8 (14.6)	2.1 (3.9)	12.1 (5.6)	62.0 (15.8)
Aβ42_het_	ff14SBonlysc_GB__–__Neck2_	34.57 (4.70)	5.93 (4.27)	15.81 (4.08)	43.69 (3.88)
Aβ42_het_	AMBER-FB15_TIP3P__–__FB_	30.77 (3.83)	9.79 (4.84)	18.41 (4.92)	41.03 (7.06)
Aβ42_het_	CHARMM36m_TIP3P*_	24.01 (4.16)	4.18 (1.82)	13.07 (4.30)	58.73 (4.03)
α-Syn_mon_	ff14SBonlysc_GB__–__Neck2_	19.60 (6.86)	22.40 (6.22)	18.18 (1.59)	39.82 (2.56)
α-Syn_mon_	AMBER-FB15_TIP3P__–__FB_	1.06 (0.56)	43.59 (1.50)	17.70 (2.33)	37.66 (1.35)
α-Syn_mon_	CHARMM36m_TIP3P*_	3.36 (1.34)	31.04 (7.98)	6.09 (0.97)	59.50 (6.19)
α-Syn_het_	ff14SBonlysc_GB__–__Neck2_	21.05 (4.74)	15.99 (5.11)	17.84 (1.34)	45.12 (4.83)
α-Syn_het_	AMBER-FB15_TIP3P__–__FB_	24.69 (2.40)	18.22 (1.74)	15.16 (2.17)	41.93 (1.86)
α-Syn_het_	CHARMM36m_TIP3P*_	18.76 (2.68)	17.12 (4.00)	12.68 (2.28)	51.45 (4.94)

a Indexes: mon—monomeric form, het—in heterodimer. Value for monomeric form of Aβ42_mon_ in CHARMM36m is taken from previous study.^51^ Values in brackets, if present, show standard deviations.

Analysis of the secondary structure of the given amino acid residues (Figure S7) shows that there are no significant changes in the tendency of given residues to form a particular type of secondary structure. For α-Syn, we did not observe large similarities to secondary structure propensities to form mostly α-helices for residues 1–60, β-sheets for 61–95, and an unstructured part for 96–140, which is established experimentally.^85^ This indicates that the monomeric structure is much different than fibrillar. Residues 45–57 and 71–82 are known to be responsible for α-Syn aggregation and its pathogenic properties.^83,86^ Our studies show that the β-structure of the latter part is disturbed upon the binding of Aβ42 (Figure S7), especially residues 72 and 74, which stopped forming β-sheets. Also, the region before and including NAC (or even residues 9–89 if connected to the membrane^87^) is responsible for forming α-helices in monomeric and tetrameric forms of α-Syn,^88^ which is disturbed upon Aβ42 binding.

While the secondary content of monomeric Aβ42 in implicit solvent simulations is similar to that in explicit solvent simulations performed in previous studies,^51^ indicating only a small overstabilization of secondary structure elements, the secondary content of monomeric α-Syn in implicit solvent simulations is significantly different from that in explicit solvent simulations. This is especially visible in the significantly larger contribution of β-structures (19.60% compared to 1.06 and 3.36%). Similarly, as for the heterodimer, CHARMM36m determines larger unstructured fragments of monomeric α-Syn than implicit and explicit solvent simulations in Amber force fields.

It should be noted that these results are very difficult to compare to experimental data because experimental studies cannot truly observe monomeric conformations due to the relatively high concentration of studied compounds necessary in such experiments, which causes rapid α-Syn aggregation.^89^ The formation of β-hairpin by residues 38–53 (by two regions rich in β-structures: 38–44 and 47–53) was found to be crucial for α-Syn aggregation in computational studies using a hybrid-resolution model.^90^ Our studies show that the first of these fragments often formed β-structures in both monomeric and heterodimeric forms, and it is involved in the interaction interface with Aβ42, suggesting similarities between binding modes of fibrils and the α-Syn–Aβ42 heterodimer.

#### 3.1.6 Similarities to Fibril Structures

Using lattice models, Li et al.^91^ showed that the fibril formation time exponentially increases with the population of the so-called fibril-prone conformation N* in the monomeric state. N* is defined as the monomer conformation in the fibril state. The relationship between the aggregation rate and the population of fibril-prone conformation has been confirmed by off-lattice coarse-grained^92^ and all-atom^93^ simulations. Therefore, it is interesting to know how the presence of a foreign chain affects the population of N* of a given chain.

We have not often detected fibril-like Aβ42 structures during the simulations. Some fibril-like conformations were mostly obtained in the first half of the simulations (not equilibrated ones), which indicates that chains are generally flexible during the simulations, but such a conformation is not energetically preferable. Although generally rare, Aβ42 more often obtained S-shape conformation rather than U-shape; however, the similarity varied depending on the (proto)fibrillar structures used as a reference within a given class. In addition, the probability of fibril-like structures decreases further in the presence of α-Syn (Tables S3 and S4). Thus, we anticipate that Aβ fibril formation slows down in the presence of α-Syn. Similar behavior, but to a lesser extent, was observed for α-Syn, which did not often form fibril-like conformations, and the Aβ42 binding decreased this probability even further (Table S5). To calculate the population of N* conformation, we took into account only residues 17–40 and 61–95 for Aβ42 and α-Syn, respectively, which are ordered in the fibril state.

#### 3.1.7 Intramolecular and Intermolecular Contacts

We have followed the time evolution of intramolecular and intermolecular contacts for both α-Syn and Aβ42 in both monomeric and heterodimeric forms (Figure S8) to observe that the fluctuation in the number of contacts is suppressed in the case of the heteromeric structure, while the average number of contacts remains similar. Our conclusions are valid for both α-Syn and Aβ42 cases. In particular, the number of intermolecular contacts in the heterodimer exhibits larger variation during the course of the simulation (Figure S7) than for intramolecular contacts. We observe that α-Syn and Aβ42 establish about 10–15 contacts on average. We can identify the most important contacts by determining the contact map of the intermolecular contacts (Figure S8). First of all, we find that contacts are well spread between different parts of the chains, suggesting that our simulation protocol offers adequate sampling across the spectrum of states. Additionally, many of the contacts have as high as 18% probability of occurring during the simulation.

**Figure 6:**
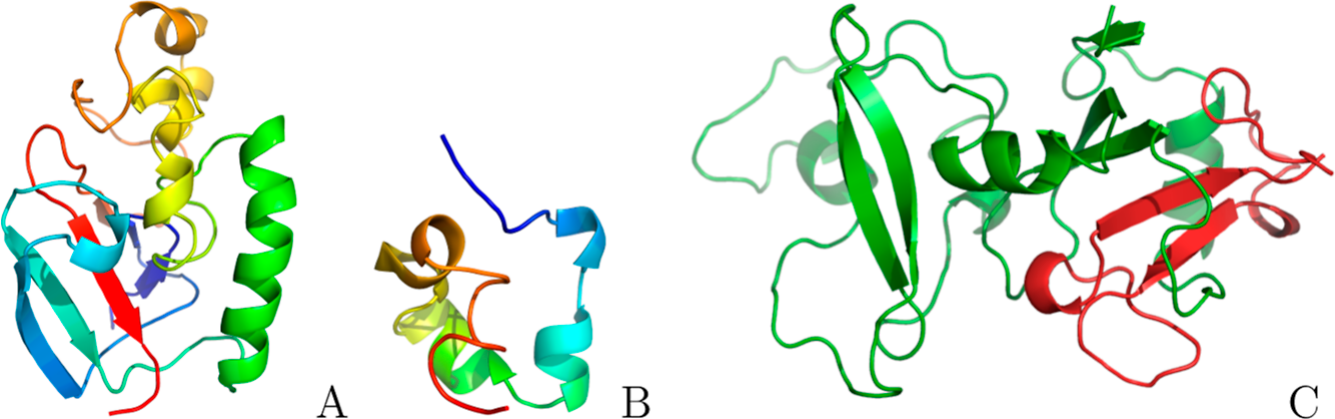
Representative structures of (A) α-Syn, (B) Aβ42, and (C) heterodimer determined from clustering of the free energy minimum basins of implicit solvent simulations, presented in cartoon representation.

We have identified the most frequent residue contacts, which are listed in Table S6. From the point of view of α-Syn, intermolecular contacts usually occur in the N-terminal domain, and even more contacts appear between the negatively charged C-terminal domain of α-Syn and Aβ42. Most contacts are established between the charged parts of the two different chains. From the point of view of Aβ42, contacts between residues 16–18 and 29–35 of Aβ42 and α-Syn appear frequently. This behavior is also reflected in the case of intramolecular contact maps when comparing the monomeric and heteromeric cases for individual chains (Figure S8). Finally, we have identified the heterodimeric structure with the highest probability based on detailed contact map and free energy map analysis, presented in Figure 6.

α-Syn forms strong interactions with Aβ42 by residues 26–28, 36–37, and 69–71 with frequencies of about 25% based on REMD simulations at approximately 300 K. These fragments align well with previous observations based on MD studies that imperfect repeats R3 (residues 31–41) and R6 (residues 68–78) play a crucial role in α-Syn aggregation and may be used as potential targets for inhibitors.^94^

### 3.2 Explicit Water Simulations of the α-Syn–Aβ42 Heterodimer

In our investigation, three out of five trajectories, as obtained with AMBER-FB15, exhibited rmsd values under 6 Å, while the other two trajectories remained just under 10 Å (Figure S9). Notably, these fluctuations predominantly originated from flexible regions in both molecules, with α-Syn, being a larger molecule, contributing more significantly. In contrast, explicit solvent simulations in CHARMM36m displayed slightly larger structural fluctuations, characterized by an average rmsd of approximately 8 Å after 700 ns, with all trajectories exhibiting similar behavior. This differed from AMBER-FB15 simulations, where two out of five trajectories presented significantly larger rmsd values than the remaining three. Both rmsd and *R*_g_ plots (Figures S9–S10) indicate stabilization of the system after 700 ns in both force fields. Therefore, our analysis focused on the range from 700 to 1000 ns, assuming convergence. This approach also helped mitigate bias arising from utilizing the representative model from implicit solvent simulations as the initial structure for explicit solvent simulations.

Analyzing fluctuations in amino acid residues from AMBER-FB15 simulations revealed that the structure of Aβ42 was considerably more stable than that of α-Syn, with only residues 22–27 displaying larger fluctuations. In α-Syn, multiple regions, particularly in the C-terminal part not involved in binding with Aβ42, exhibited significant fluctuations. This contrasts results obtained from implicit solvent REMD simulations, where fluctuations in the heterodimer, especially in α-Syn, were larger than in monomeric structures. This discrepancy is reasonable due to the presence of multiple binding modes in the complex, causing variations in different parts of the molecules. Intriguingly, RMSF plots for CHARMM36m closely resembled those for AMBER-FB15, indicating similar regions of high flexibility in the heterodimer. Explicit solvent MD simulations for both force fields slightly decreased the compactness of the heterodimer, more pronounced in CHARMM36m, where an increase in *R*_g_ by about 1–2 Å was observed. This behavior aligns with the generally lower compactness of conformations observed in this force field.^46^

The most notable distinction between implicit and explicit solvent simulations emerged in monomeric α-Syn. Implicit solvent simulations significantly overpredicted the β-content, whereas explicit solvent simulations for the heterodimer in both solvents showed no significant changes in secondary structure distribution. While AMBER-FB15 exhibited a slight tendency to increase the content of α-helices and β-sheets, resulting in a small decrease in unstructured fragments compared to implicit solvent simulations, CHARMM36m displayed the opposite behavior, slightly increasing the percentage of unstructured fragments of the heterodimer by a small reduction in β-sheets and turns (Table 2). Despite some conformational fluctuations observed in explicit solvent simulations, they did not significantly impact the secondary structure content of the heterodimer, which remained stable during explicit solvent simulations, maintaining an average similar to implicit solvent simulations (Figure S7).

In summary, both force fields, employing the explicit solvent model, demonstrated slightly larger flexibility and less compactness of the heterodimer. However, the complex remained stable in all trajectories, confirming the high stability of the predicted model.

#### 3.2.1 Aβ42 Increases α-Syn Aggregation Propensity

It has been demonstrated that the β-content of the monomer determines the propensity for aggregation: the higher the β-content in the monomeric state, the faster aggregation occurs.^95^ To investigate whether the presence of Aβ42 accelerates α-Syn aggregation, we compare the β-contents of α-Syn in the absence and presence of Aβ42. In implicit water simulations with ff14SBonlysc_GB–Neck2_, the β-content of α-Syn monomer (19.60 ± 6.86) is similar to that in the heterodimer (21.05 ± 4.74) (Table 2). However, the situation changes significantly in explicit water simulations. The presence of Aβ42 increases the β-content from 1.06 ± 0.56 to 24.69 ± 2.40 (AMBER-FB15_TIP3P–FB_) and from 3.36 ± 1.34 to 18.76 ± 2.68 (CHARMM36m_TIP3P*_). The impact of Aβ42 on α-Syn aggregation is not observed in implicit water models, further showing a significant overestimation of the stability of monomeric α-Syn. However, the β-content of the α-Syn-Aβ42 in the implicit solvent simulations falls between the observed values for explicit solvent simulations in AMBER15-FB and CHARMM36m. On the other hand, explicit water models capture the experimental observation of enhanced α-Syn aggregation by Aβ42.

#### 3.2.2 Prediction of Amino Acid Residues Involved in Binding Interface

The contact map reveals that there are several interactions between residues, which are responsible for the binding (Figure S11). Phe19, Phe20, Ile31, Ile32, Gly37, and Gly38 from Aβ42 are forming stable contacts with Val26, Ala27, Glu28, Gly36, Val37, Tyr39, Val40, and Gly47 from α-Syn, which form the hydrophobic core of the heterodimer (Figure S10). This results in strong binding associated with binding free energy, Δ*G* = −48.26 ± 9.74 kcal/mol, as obtained by using the MM-PBSA method averaged over five trajectories.

To confirm whether selected residues are strongly interacting at the interface and are not just close due to the accidental proximity, we also calculated the binding free energies per residue (Table 3) using the most representative structure of the heterodimer in the HawkDock server.^66^ The performed analysis shows that residues from Aβ42 interact stronger than the ones from α-Syn and there is an overall good agreement between the distance prediction from the trajectory and the energy prediction from the representative model of the residues involved at the interaction interface. It can be seen that residues, which are involved in interaction with partner molecule, are characterized by much lower conformational flexibility than surrounding residues (Figure S12), which confirms that binding stabilizes the interacting regions of both molecules.

**Table 3:** Residues in the α-Syn–Aβ42 Heterodimer Model That Interact Strongly (Binding Free Energy <−2 kcal/mol)

strongly interacting residues			
α-Syn		Aβ42	
Res	dG	Res	dG
VAL 37	–5.37	ILE 32	–8.29
VAL 26	–4.22	PHE 20	–7.12
TYR 39	–3.74	PHE 19	–7.11
ALA 27	–3.66	ILE 31	–4.52
THR 33	–3.22	VAL 18	–3.96
GLU 28	–2.68	VAL 12	–3.86
ALA 30	–2.68	VAL 36	–3.82
LYS 45	–2.61	GLY 38	–3.58
GLN 62	–2.22	LEU 17	–3.31
ALA 29	–2.05	LYS 28	–3.16
		ALA 21	–2.8
		ARG 5	–2.3
		GLN 15	–2.17

#### 3.2.3 Comparison of Aβ42 and α-Syn Homo- and Heterodimer Binding Affinities

**Figure 7:**
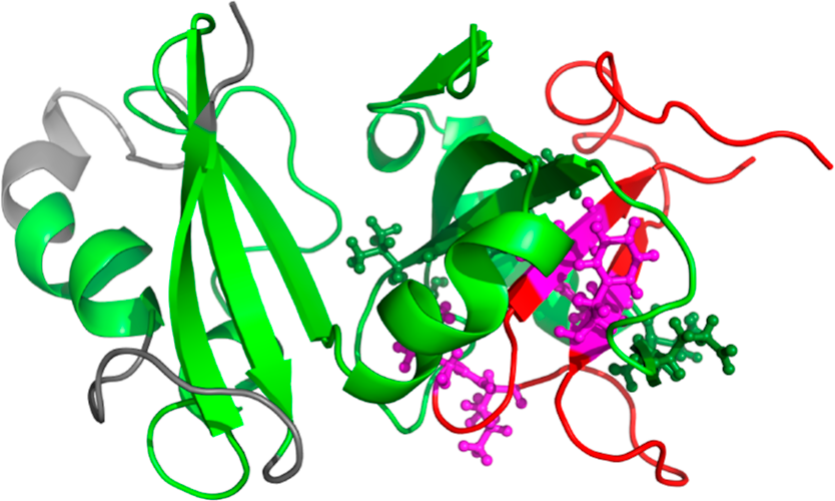
Cartoon representation of the most probable heterodimer complex determined from all-atom implicit MD simulations followed by explicit solvent MD simulation with residues forming stable interactions represented by ball-and-stick representation. In addition, highly flexible fragments are marked with gray color.

A comparison of the predicted binding free energies for representative structures of the heterodimer to those of Aβ42 dimeric models from previous studies (Table 4) indicates that heterodimers are more stable than the respective homodimers. At the same time, interactions between dimeric α-Syn are even stronger. The same relation is showed by a much faster analysis of single representative snapshots, performed by HawkDock (Table 4). It should be noted that relative values, rather than absolute ones, should be compared when molecular modeling methods are used. This result is in agreement with the experimental observation that monomeric α-Syn, contrary to fibrillar forms, inhibits secondary nucleation of Aβ42 fibrils by the formation of strong interactions between molecules.^32^ This allows us to propose with confidence the most probable heterodimer structure, which is shown in Figure 7 (the PDB structure is provided as Supporting Information to the article).

**Table 4:** Binding Free Energies [kcal/mol] Predicted by Using the MM-PBSA (MM) Analysis for the Trajectories with Standard Deviations and the HawkDock (Hawk) Server for Representative Models

	Aβ42 dimer		α-Syn–Aβ42		α-Syn dimer	
	MM	Hawk	MM	Hawk	MM	Hawk
1	–9.90 ± 5.22	–43.38	–48.26 ± 9.74	–113.15	–61.78 ± 35.73	–176.54
2	–22.60 ± 3.80	–52.07			–80.53 ± 13.96	–176.46
3	–20.11 ± 7.01	–58.17			–28.09 ± 12.27	–80.97

Numerous studies have demonstrated that certain amino acid residues or fragments of the Aβ structure play a crucial role in its propensity to form aggregates. Maity and colleagues^96^ have shown that Aβ(14–23) forms a hairpin, which, upon aggregation, resembles full-size Aβ42 fibrils. This effect is further observed by Sun et al. as a key factor in the formation of beta-barrels in the early stages of aggregation in AD.^97^ In another study, Khaled et al.^98^ found that residues 15–20 in Aβ may form contacts, especially in truncated variants lacking C-termini [e.g., Aβ(1–28)]. However, in full-length Aβ42, the key to fibrillization lies in the hydrophobic interactions between the central hydrophobic core (residues 16–22) and the C-terminal region (residues 30–42). Other regions (1–15 and 21–28) lack stable secondary structures and primarily serve as hinges. This finding is corroborated by the work of Itoh et al.,^99^ who demonstrated that the formation of two antiparallel beta-sheets between the C-terminus and the center of the peptide is crucial for aggregate formation. The formation of a beta-hairpin also plays a crucial role in dimerization and higher oligomerization of Aβ(29–42) variants, indicating the important role of the C-terminal part of Aβ42.^100,101^ The fact that the β-hairpin promotes aggregation is also consistent with the N*-theory showing that the faster the fibril formation, the higher the propensity of the fibril-prone structure.^91,92,95^ However, this interaction is not purely hydrophobic, as Arg5 electrostatically stabilizes the molecule, forming interactions with residues such as E22 and K28.^99^ This effect was also observed in MD simulations by Huy et al.,^60^ showing that Arg5 forms salt bridges with Asp1, Glu22, and Asp23, stabilizing the N-terminal region of Aβ42, which can be disrupted in the presence of copper ions.

Our MD simulations of a heterodimer show that Aβ42 fragments 16–19 and 29–34 (Table S6) are the most involved in forming interactions with α-Syn, which quite accurately fit within the hairpin regions in Aβ42 peptides and oligomers, as shown by other studies.^98,99^ They also agree with DMD-predicted regions as hotspots for the α-Syn–Aβ42 interaction, which cover residues 31–60 (the second half of the N-terminal domain) and 61–95 (NAC domain), and residues 10–21 and 31–42 in α-Syn and Aβ42, respectively.^102^ Therefore, hydrophobic interactions are also important for the formation of an α-Syn–Aβ42 heterodimer. There are residue ranges for beta-structures in a heterodimer (Figure S8), similar to the Aβ42 homodimers; however, we did not find Arg5 to be important in the formation of contacts with α-Syn. It should be noted that although Chau and Kim found that α-Syn monomers and oligomers promote oligomerization of Aβ42 most likely through binding or coassembly, various parts of Aβ42 are involved in interactions with α-Syn, strongly depending on their conformations.^103^

## 4 Conclusions

In this study, we pursued two primary objectives: (i) investigating the interactions between intrinsically disordered monomeric α-Syn and Aβ42 and (ii) assessing the capabilities of implicit solvent simulations in modeling monomeric and heterodimeric systems with disordered characteristics. To achieve this, extensive implicit solvent MD simulations were conducted for both α-Syn and Aβ42, along with their complex, and the results were compared with explicit solvent simulations. Our analysis revealed that the selected all-atom force field with an implicit solvent model (AMBER ff14SBonlysc with the GB-Neck2 model) tended to generate overly compact and ordered structures, particularly noticeable in the case of monomeric α-Syn. However, for the α-Syn–Aβ42 heterodimer, the most probable structure remained stable in both implicit and explicit solvent simulations using AMBER-FB15 and CHARMM36m force fields.

To identify the most stable heterodimer, we conducted a thorough analysis of relevant properties for both monomeric and heteromeric structures. Our findings indicated that the binding of molecules significantly influences their mobility and flexibility. Notably, α-Syn exerts a greater impact on the conformation of Aβ42 than vice versa, attributed to the hydrophobic nature of interchain interactions. Emphasizing the critical role of water models in modeling protein aggregation, we highlighted that the impact of Aβ42 on the aggregation propensity of α-Syn is accurately captured by explicit water models but not by implicit ones. Further research is required to investigate whether this conclusion holds for other scenarios. Comparing the representative α-Syn–Aβ42 heterodimer with Aβ42 and α-Syn homodimer models revealed that it binds approximately two times stronger than Aβ42 dimeric structures but weaker than the α-Syn homodimer. This suggests that α-Syn and Aβ42 can indeed form stable complexes, potentially serving as seeds for fibril structures and competing with the Aβ42 aggregation process. Our MD simulations of the α-Syn–Aβ42 heterodimer reveal that Aβ42 fragments 16–19 and 29–34 are the most involved in forming interactions with α-Syn, aligning with the β-hairpin regions in Aβ42 peptides and oligomers identified in previous studies.^100,101^ These findings also agree with DMD-predicted hotspots for the α-Syn–Aβ42 interaction, covering residues 31–60 and 61–95 in α-Syn and residues 10–21 and 31–42 in Aβ42.

Additionally, we demonstrated the efficiency of using single representative structures for estimating the binding interface and energies, providing results consistent with the more computationally expensive MM-PBSA method across multiple trajectories. Finally, we share the PDB structure of the most probable α-Syn–Aβ42 heterodimer, which can be used to further examine the structure and dynamics and design potential inhibitors.

## Supporting Information Available

Details of the simulations, temperatures used in REMD, fibril-prone Aβ and α-Syn structures data, interacting residues during REMD simulation, rmsd to experimental structures for α-Syn and Aβ42, free energy maps for α-Syn and Aβ42, RMSF of α-Syn and Aβ42 as monomers and in the heterodimer, differences of SASA values upon binding for α-Syn and Aβ42, secondary structure per residue for α-Syn and Aβ42 in implicit and explicit solvent simulations, time evolution of intermolecular contacts between α-Syn and Aβ42, contact maps of α-Syn and Aβ42 and their intermolecular contacts, rmsd and *R*_g_ of α-Syn and Aβ42 and the heterodimer during explicit solvent simulations of the α-Syn and Aβ42, and contact map from explicit solvent simulation (PDF)
PDB structure of the most stable heterodimer (PDB)
Initial and final protein conformations (ZIP)

## Supplementary Material

jp4c00503_si_001.pdf

jp4c00503_si_002.pdb
