# Supplementary material for: Exploring Structural Insights of Aβ42 and α-Synuclein Monomers and Heterodimer: A Comparative Study Using Implicit and Explicit Solvent Simulations
Source: J Phys Chem B. 2024 May 3;128(19):4655–69. doi: 10.1021/acs.jpcb.4c00503 (PMC11103699; doi:10.1021/acs.jpcb.4c00503)
Supplement: Supplementary file 1 — jp4c00503_si_001.pdf [file jp4c00503_si_001.pdf]

Supporting Information: Exploring Structural Insights of Aβ42 and α-Synuclein Monomers and Heterodimer: A Comparative Study Using Implicit and Explicit Solvent Simulations

Yuliia Varenyk,[†,‡] Panagiotis E. Theodorakis,[†] Dinh Q.H. Pham,[†] Mai Suan Li,[†] and Paweł Krupa[*,†]

†*Institute of Physics Polish Academy of Sciences, Al. Lotnikow 32/46, 02-668, Warsaw, Poland*

‡*Department of Theoretical Chemistry, University of Vienna, Vienna 1090, Austria*

E-mail: pkrupa@ifpan.edu.pl

Table S1: Types and details of simulations performed in this study along with purpose of given simulation type: REMD simulations in implicit solvent of Aβ42 and α-Syn monomers and their heterodimer; folding conventional MD simulations of α-Syn in explicit solvent; stability determination of Aβ42-α-Syn heterodimer in explicit solvent conventional MD simulations; conventional MD simulations to estimate binding affinity of Aβ42 and α-Syn dimers. Numbers in the first column corresponds to the input and output files attached in the SI file.

No	System	Purpose	Initial structure	Force field	Solvent	Simulation type	Trajectories	Total time [µs]	Temperature [K]
1.	Aβ42	Folding	Previous study [1]	Amber ff14SBonlysc	Implicit GB-Neck2	REMD	20*4000ns	80	281-512.19
2.	α-Syn	Folding	1XQ8	Amber ff14SBonlysc	Implicit GB-Neck2	REMD	20*2000ns	40	281-512.19
3.	Aβ42-α-Syn	Folding	Previous study [1] + 1XQ8 semidocked	Amber ff14SBonlysc	Implicit GB-Neck2	REMD	20*1150ns	23	281-512.19
4.	α-Syn	Folding (big box)	1XQ8	AMBER-FB15	Explicit TIP3P-FB	cMD	1*100ns	5	300
	α-Syn	Folding (smaller box)	Final structure from above MD	AMBER-FB15	Explicit TIP3P-FB	cMD	3*1667ns	5	300
5.	α-Syn	Folding (big box)	1XQ8	CHARMM36m	Explicit TIP3P*	cMD	3*1667ns	5	300
	α-Syn	Folding (smaller box)	Final structure from above MD	CHARMM36m	Explicit TIP3P*	cMD	3*1667ns	5	300
6.	Aβ42-α-Syn	Stability verification	representative from REMD	AMBER-FB15	Explicit TIP3P-FB	cMD	5*1000ns	5	300
7.	Aβ42-α-Syn	Stability verification	representative from REMD	CHARMM36m	Explicit TIP3P*	cMD	5*1000ns	5	300
8.	α-Syn-α-Syn	Binding energy estimation	Previous work [2]	AMBER-FB15	Explicit TIP3P-FB	cMD	3*5*50ns	0.75	300
9.	Aβ42-Aβ42	Binding energy estimation	Previous work [3]	AMBER-FB15	Explicit TIP3P-FB	cMD	3*5*50ns	0.75	300

Table S2: Temperature of each replica in our REMD simulations. In total 20 replicas was used for each simulation. This distribution of temperatures has been produced by using the temperature generator for remd simulations.[4,5]

Replica id	Temperature [K]	Replica id	Temperature [K]
1	281.00	11	389.68
2	290.73	12	402.06
3	300.69	13	414.74
4	310.89	14	427.24
5	321.35	15	440.55
6	332.06	16	454.19
7	343.04	17	468.16
8	354.29	18	482.48
9	365.80	19	497.16
10	377.59	20	512.19

Table S3: Fibril-prone Aβ structures observed in simulations or structures with less than **4** Å deviated from fibril-extracted peptides calculated for 17–40 amino-acid residues. Data show number of fibril-like snapshots in full trajectory and in parentheses are those in the second half of trajectory.

Reference	Aβ alloform	Shape	Aβ	Aβ in complex
2MXU	Aβ_{42}	S	2(1)	0(0)
2NAO	Aβ_{42}	S	41(7)	0(0)
5KK3	Aβ_{42}	S	28(4)	0(0)
5OQV	Aβ_{42}	L-S	68(14)	0(0)
2M4J	Aβ_{40}	U	6(3)	0(0)
2BEG	Aβ_{42}	U	0(0)	0(0)
2LMN	Aβ_{40}	U	0(0)	0(0)

Table S4: Fibril-prone Aβ structures observed in simulations or structures with less than **5** Å deviated from fibril-extracted peptides calculated for 17–40 amino-acid residues. Data show the number of fibril-like snapshots in the full trajectory and in parenthesis those in the second half of trajectory.

Reference	Aβ alloform	Shape	Aβ	Aβ in complex
2MXU	Aβ_{42}	S	500(168)	41(0)
2NAO	Aβ_{42}	S	4332(2289)	1056(698)
5KK3	Aβ_{42}	S	2771(1161)	57(0)
5OQV	Aβ_{42}	L-S	2070(822)	6(1)
2M4J	Aβ_{40}	U	881(310)	23(0)
2BEG	Aβ_{42}	U	118(55)	114(2
2LMN	Aβ_{40}	U	62(12)	32(32)

Table S5: Fibril-prone α-Syn structures observed in simulations or structures with less than **6** Å and **7** Å deviated from fibril-extracted peptides calculated for 61–95 amino-acid residues (NAC part). Data show the number of fibril-like snapshots in the full trajectory and in parenthesis those in the second half of trajectory.

Reference	α-Syn		α-Syn in complex	
	6 Å	7 Å	6 Å	7 Å
2N0A	1 (0)	108 (28)	0	8 (0)
6CU7	0	165 (139)	0	98 (0)
6RT0	0	116 (0)	0	1 (0)

Table S6: Residue pairs in the α-Syn–Aβ42 heterodimer that interact during the simulation.

Interacting residue pairs			
α-Syn	Aβ42	α-Syn	Aβ42
42 (Ser)	39 (Val)	54 (Thr)	17 (Leu)
54 (Thr)	18 (Val)	60 (Lys)	11 (Glu)
97 (Lys)	34 (Leu)	108 (Pro)	32 (Ile)
109 (Gln)	31(Ile)	110 (Glu)	16 (Lys)
111 (Gly)	30 (Ala)	113 (Leu)	29 (Gly)
128 (Pro)	19 (Phe)		

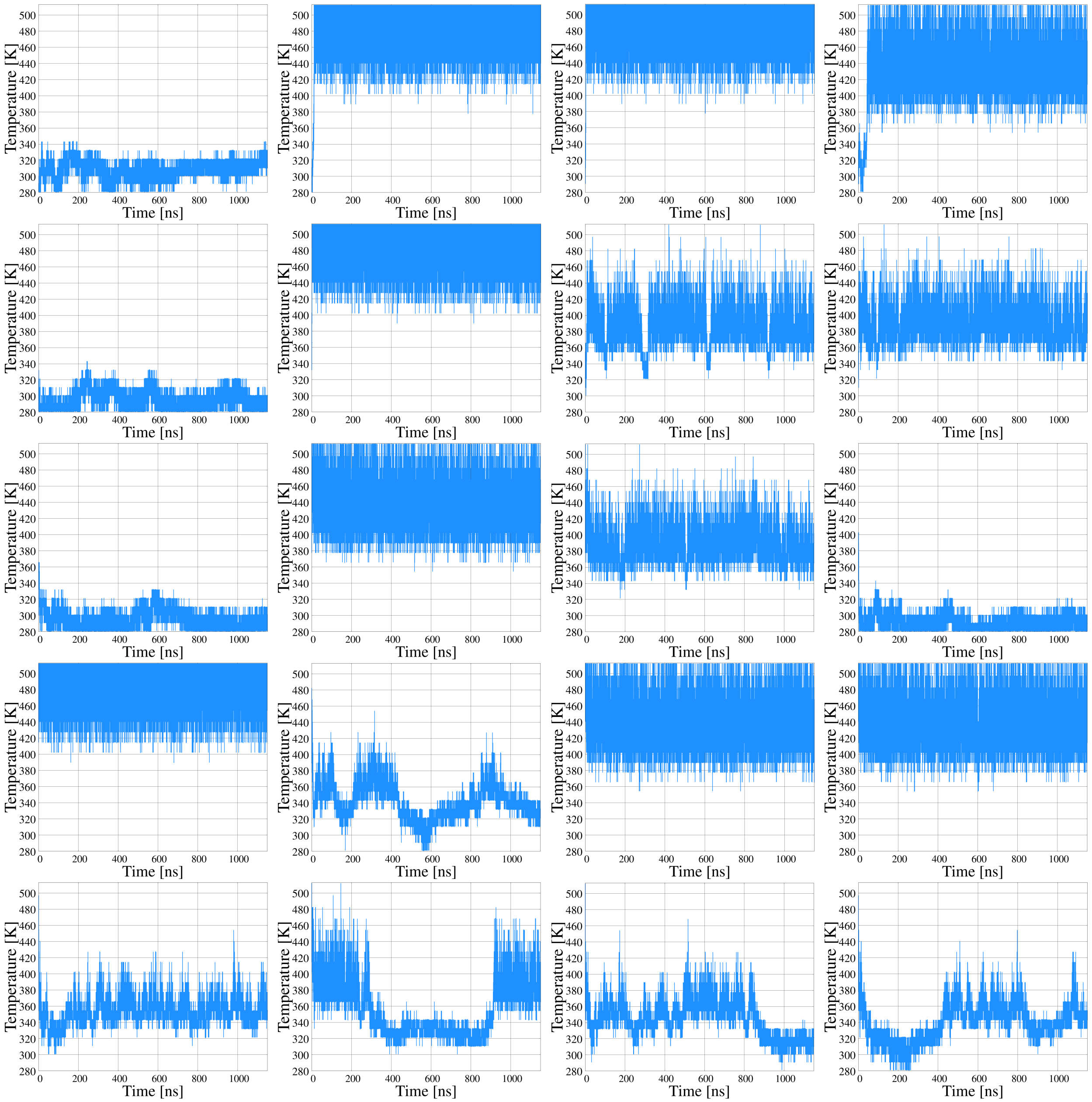

Figure S1: Temperature walk during REMD simulation of the α-Syn–Aβ42 heterodimer complex - one plot represents a single trajectory.

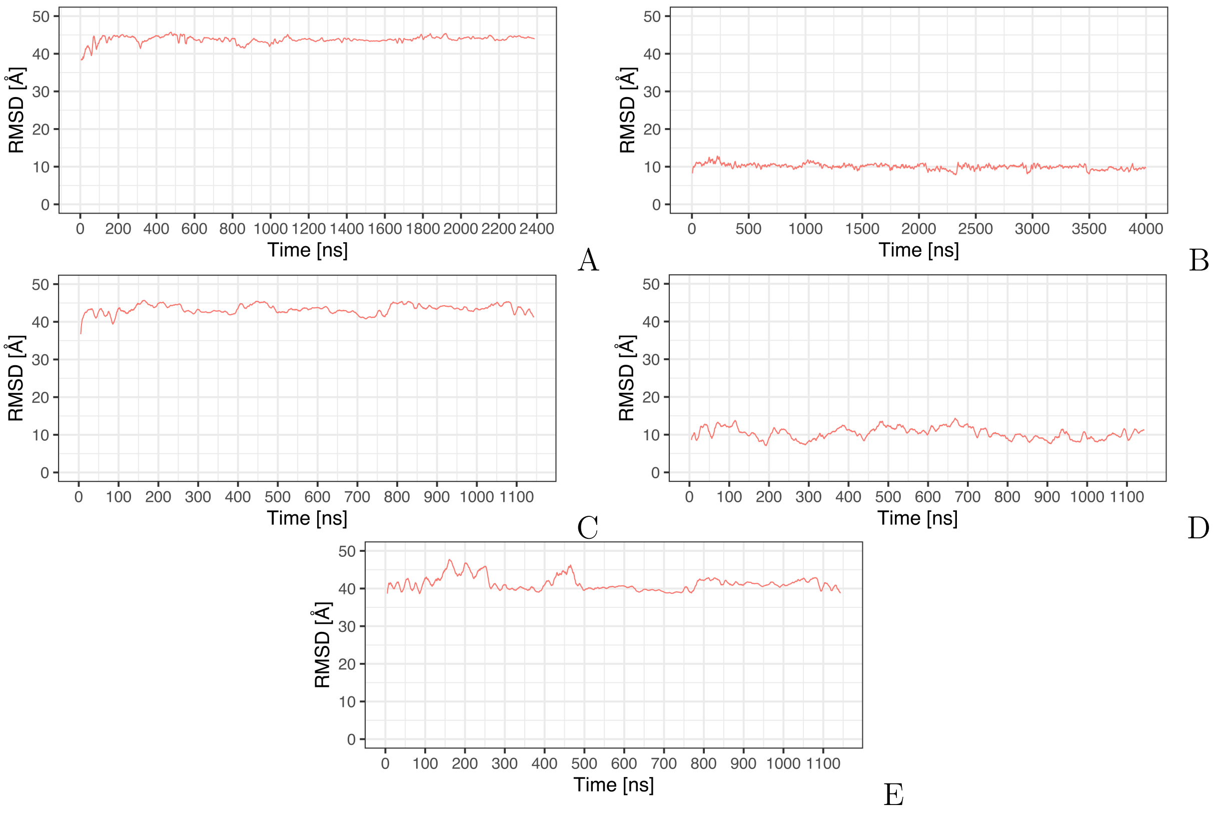

Figure S2: Time evolution of RMSD during implicit water simulations of α-Syn and Aβ42 as monomers (A, B), in the heterodimer (C, D), and the α-Syn–Aβ42 heterodimer complex (E) with respect to the initial structures.

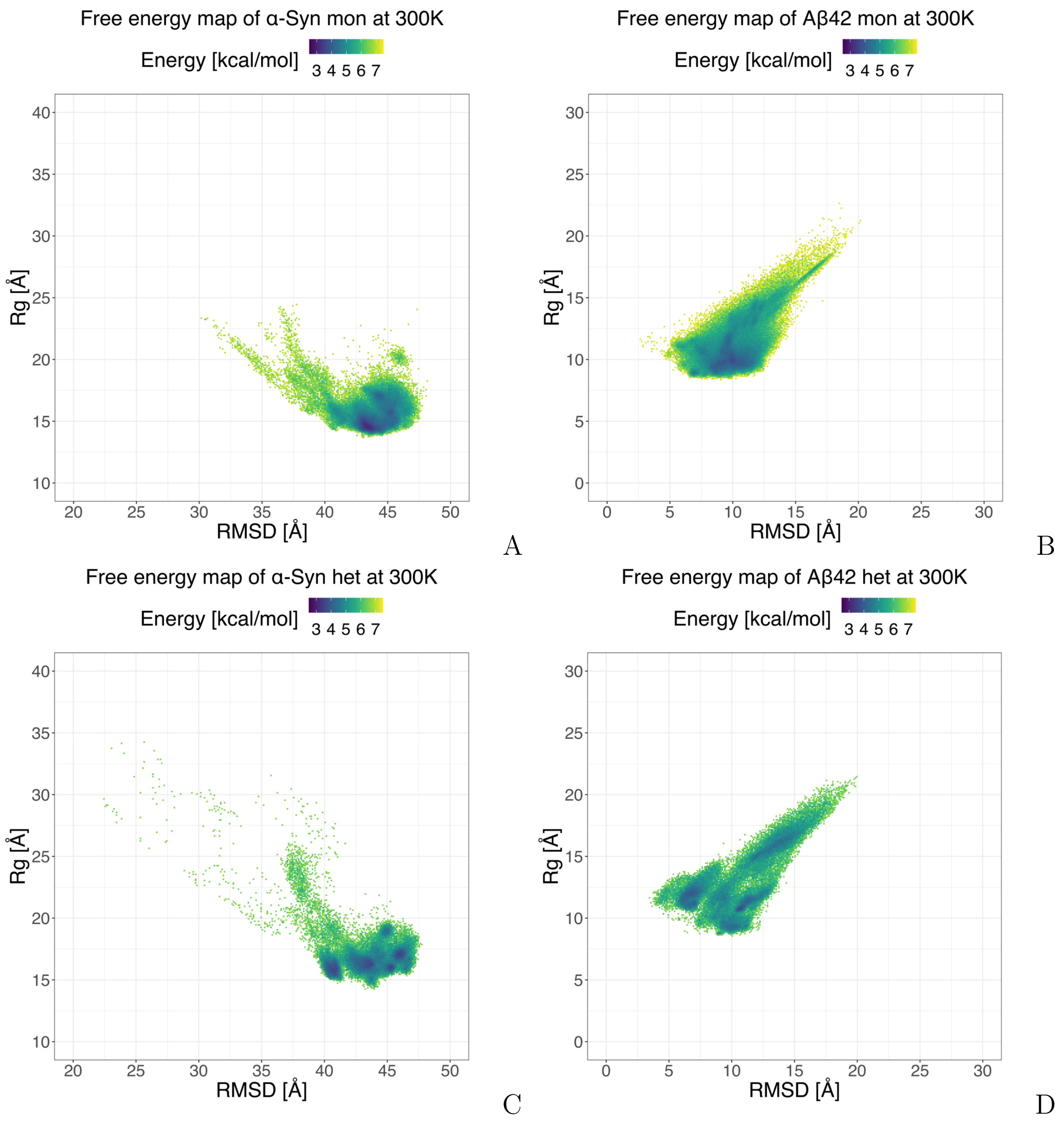

Figure S3: Free energy map as a function of radius of gyration, R_g, and the RMSD (in respect to the initial structures) of α-Syn and Aβ42 as monomers (A, B), and in the heterodimer (C, D).

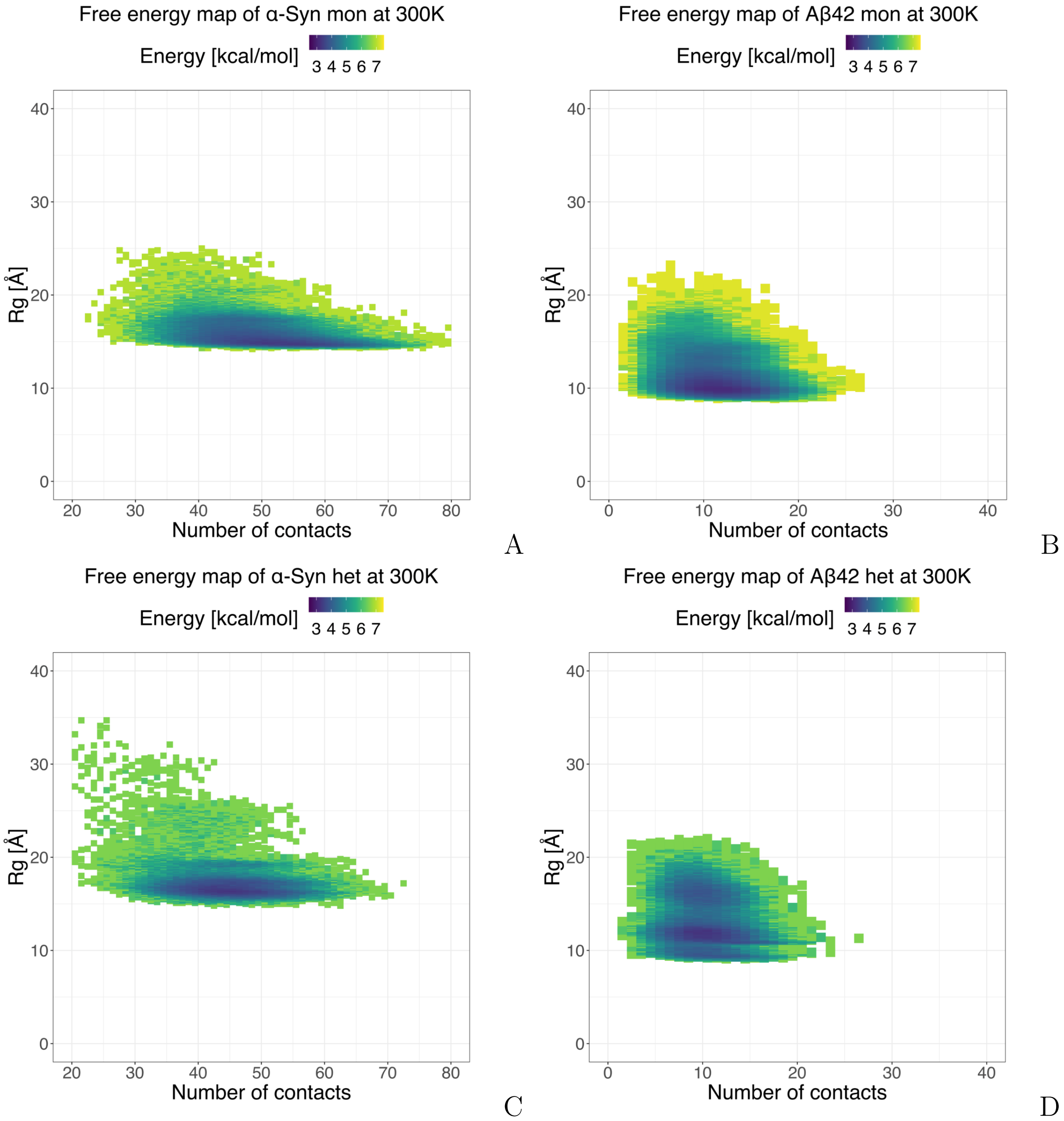

Figure S4: Free energy map as a function of the radius of gyrations, R_g, and the number of contacts for α-Syn and Aβ42 as monomers (A, B), and in the heterodimer (C, D).

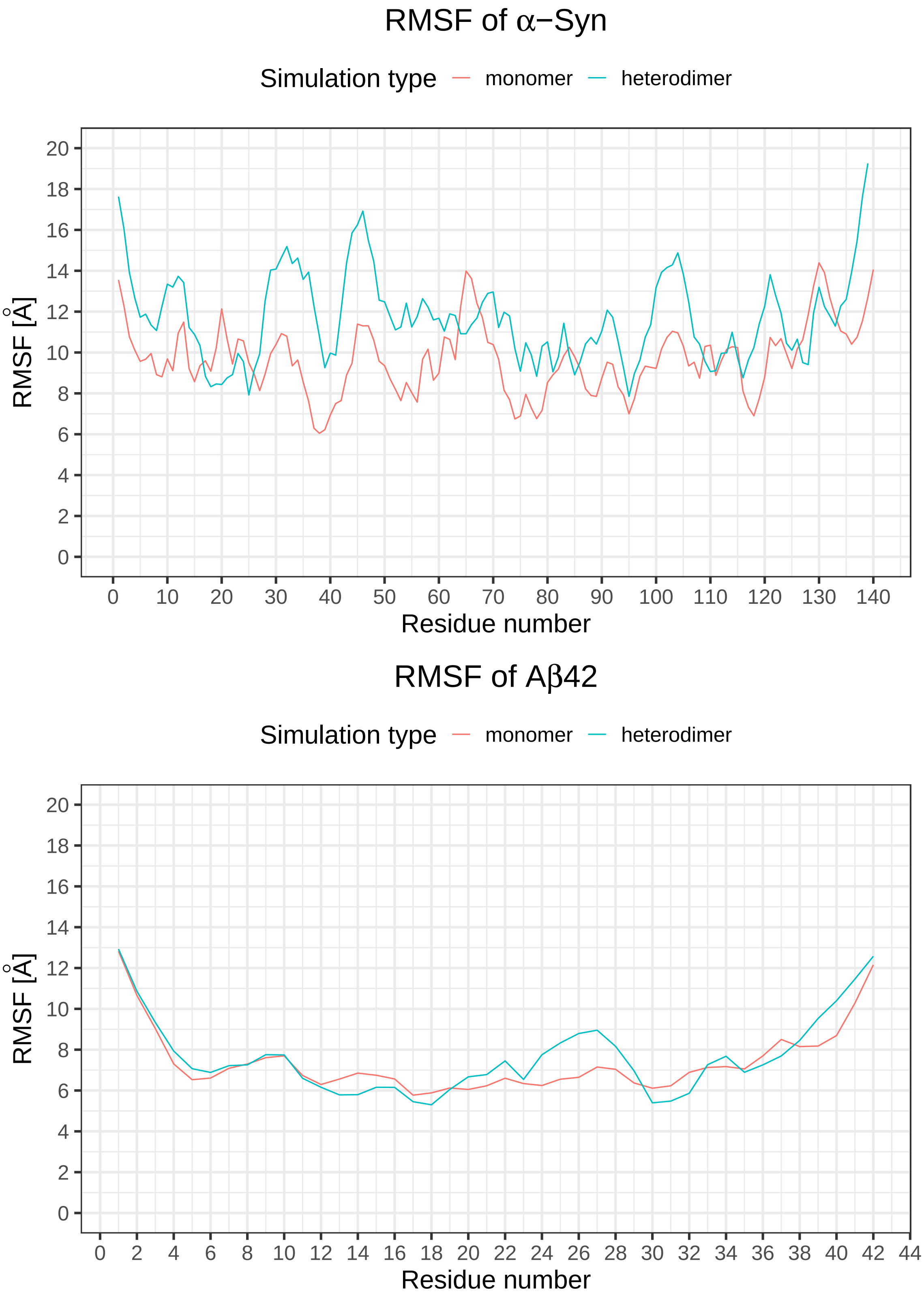

Figure S5: RMSF of α-Syn and Aβ42 as monomers and in the heterodimer as indicated. The reference structure here is the average structure over the equilibrated part of the trajectory.

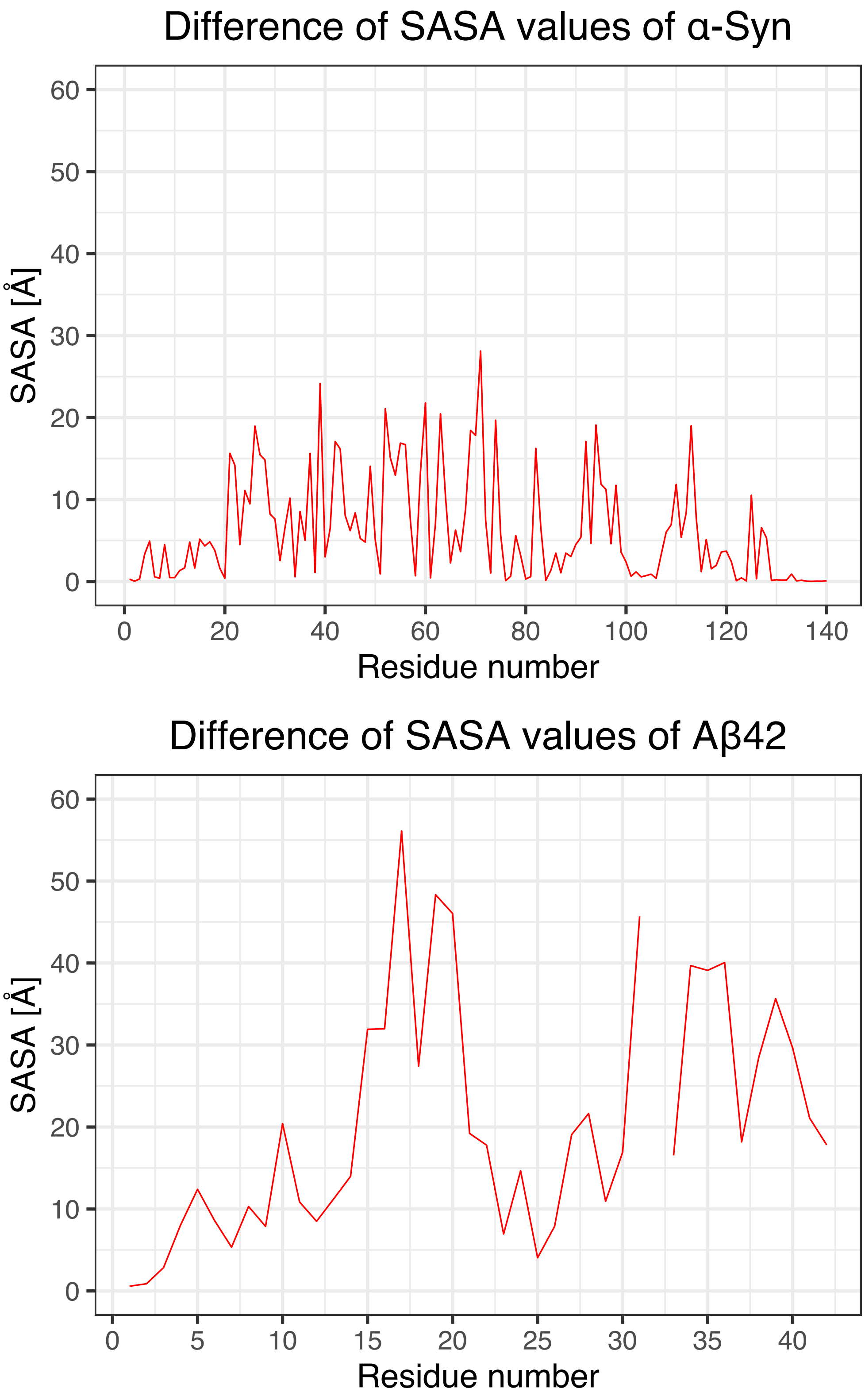

Figure S6: Difference of per residue SASA values for α-Syn (upper panel) and Aβ42 (lower panel) from simulations in heterodimer system (SASA value with second molecule stripped - SASA value with second molecule present (non-stripped)).

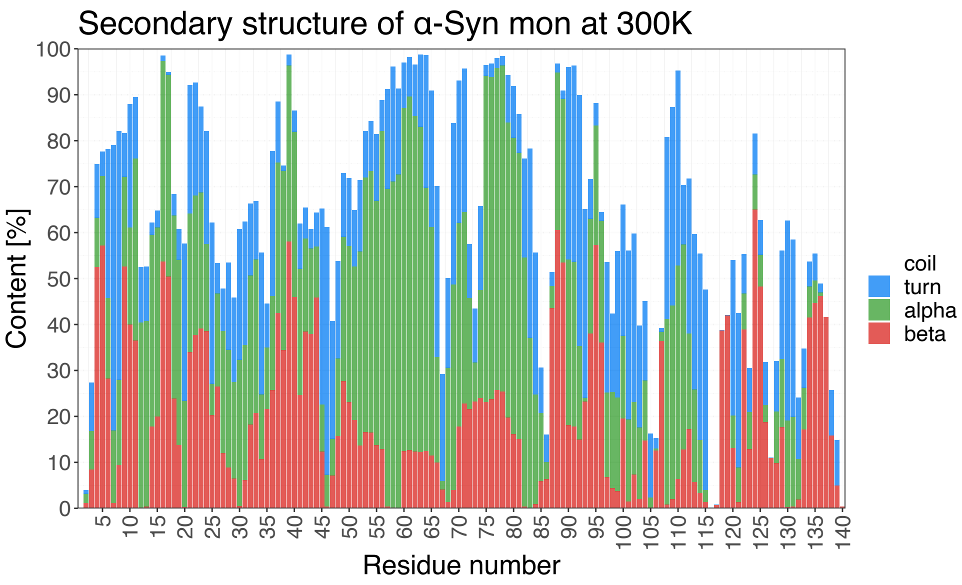

Secondary structure of α-Syn mon at 300K
Content [%]
Residue number
coil
turn
alpha
beta

A

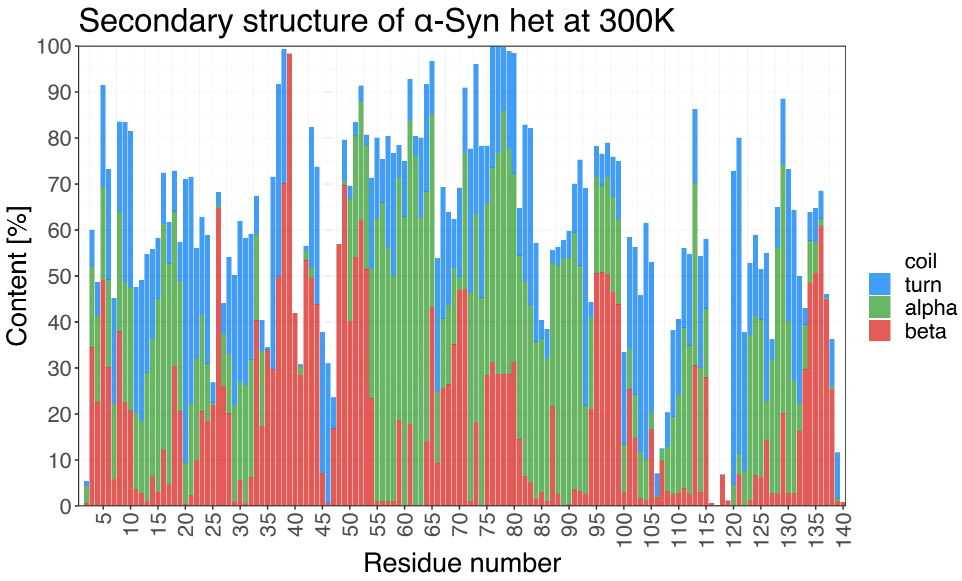

Secondary structure of α-Syn het at 300K
Content [%]
Residue number
coil
turn
alpha
beta

B

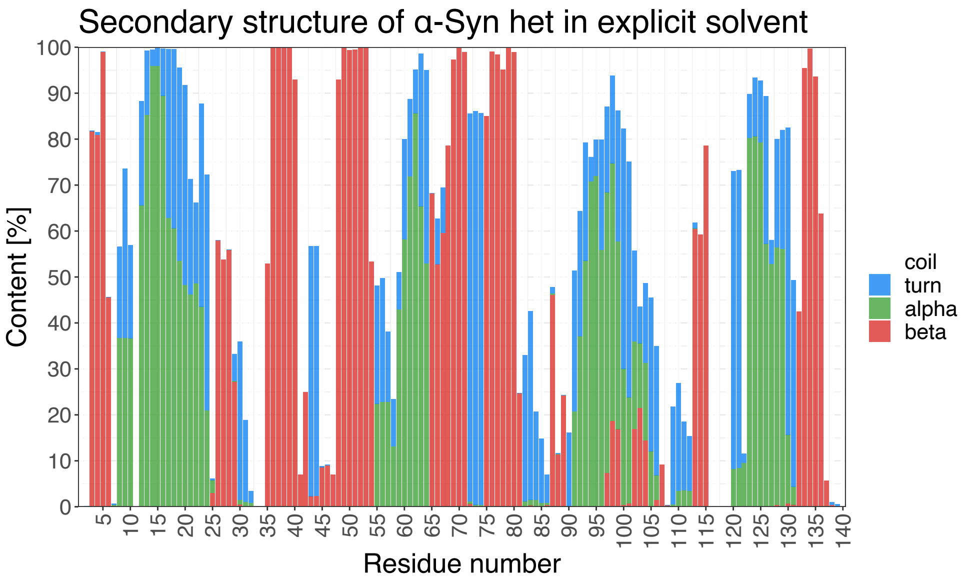

Secondary structure of α-Syn het in explicit solvent
Content [%]
Residue number
coil
turn
alpha
beta

C

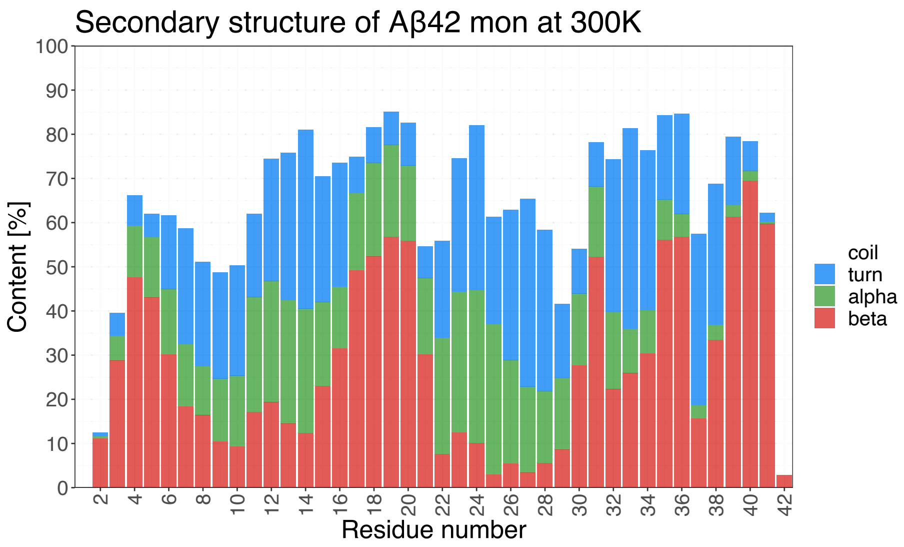

D

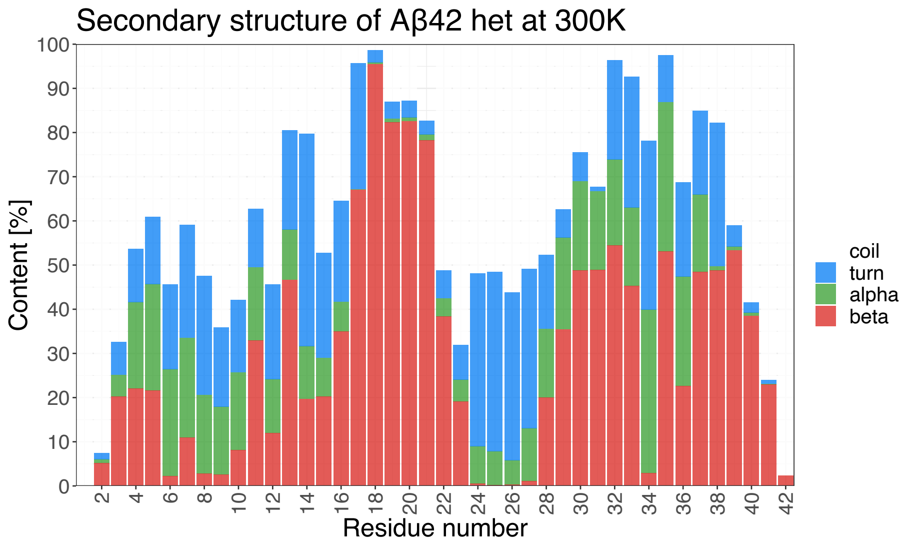

E

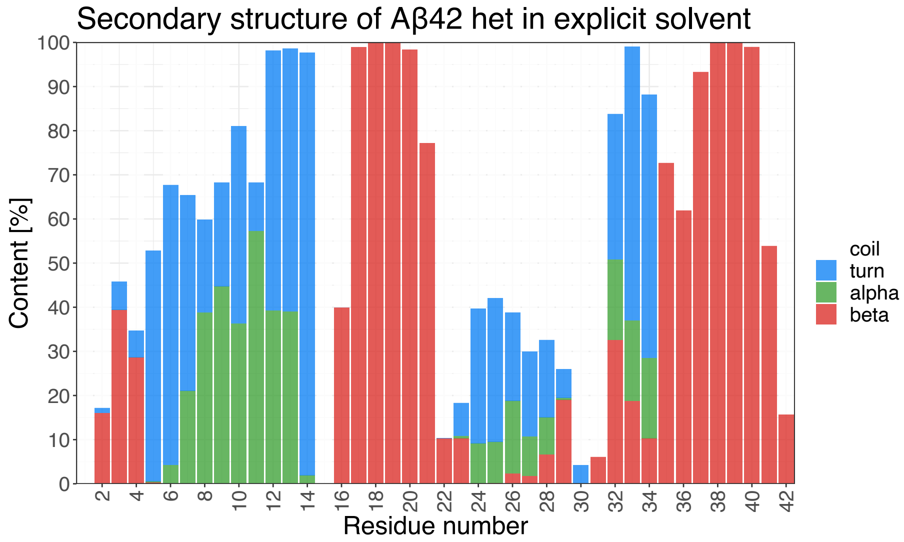

F

Figure S7: Secondary structure per residue for α-Syn simulated as monomer (A), heterodimer (B), and from explicit solvent simulations (C), and Aβ42 as monomer (D), heterodimer (E), and from explicit solvent simulations (F).

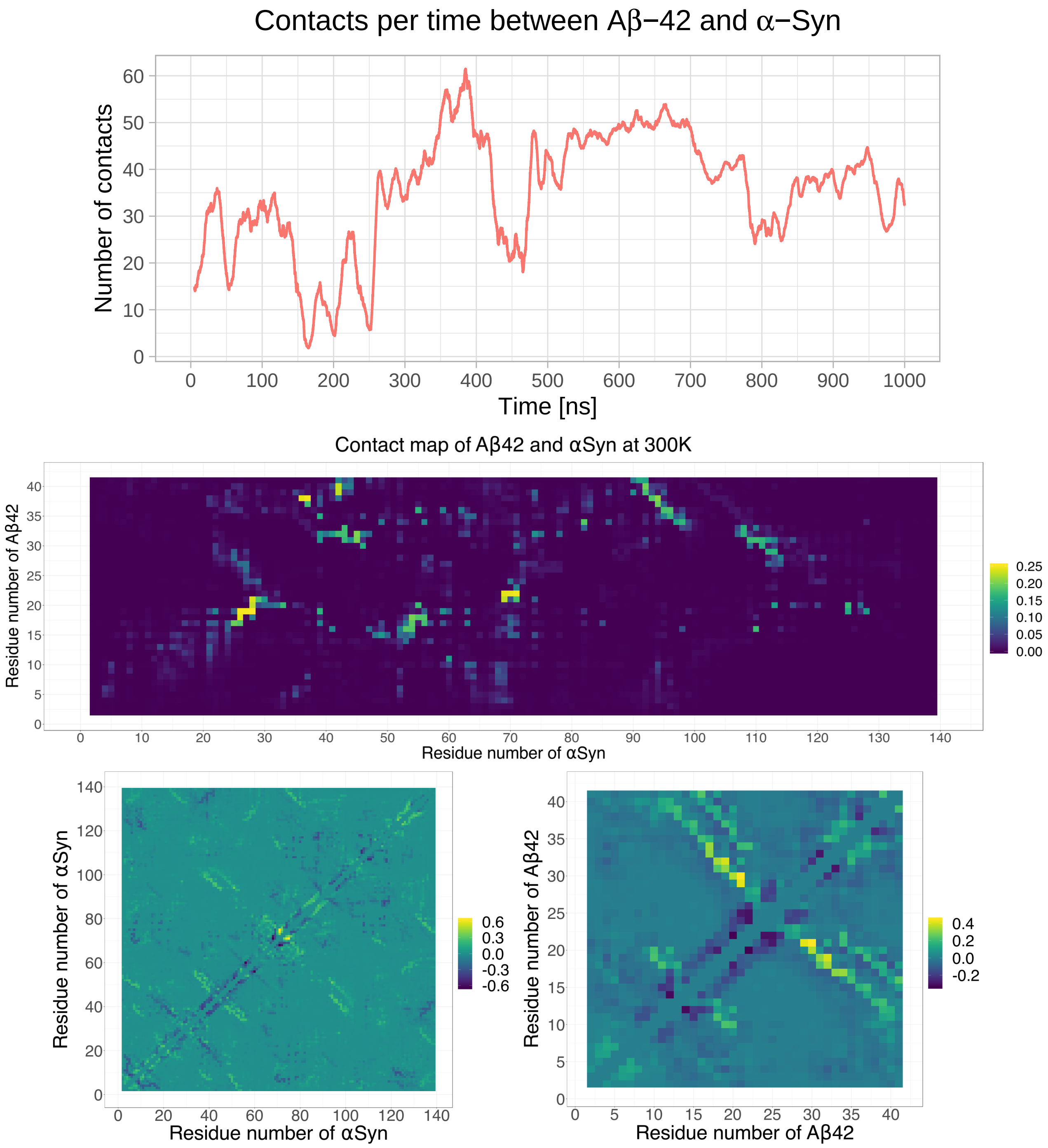

Figure S8: The time evolution of intermolecular contacts between α-syn and Aβ42 (upper panel), and the contact map between α-Syn and Aβ42 (middle panel). The contact maps of α-Syn and Aβ42 for the monomeric cases are shown in the lower panel. The colour map indicates the probability of a particular contact between α-Syn and Aβ42.

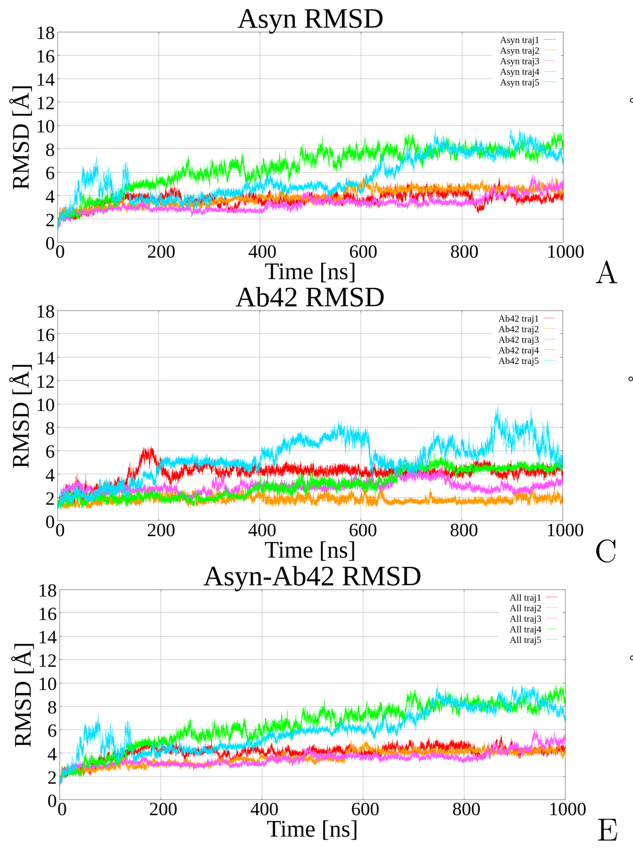

Figure S9: Plot of the RMSD values for (A-B) α-Syn, (C-D) Aβ42, and (E-F) heterodimer during explicit water simulations of the α-Syn – Aβ42 complex in AMBER-FB15 (A, C, E) and CHARMM36m (B, D, F) calculated in respect to the initial structure (representative structure from the REMD simulation).

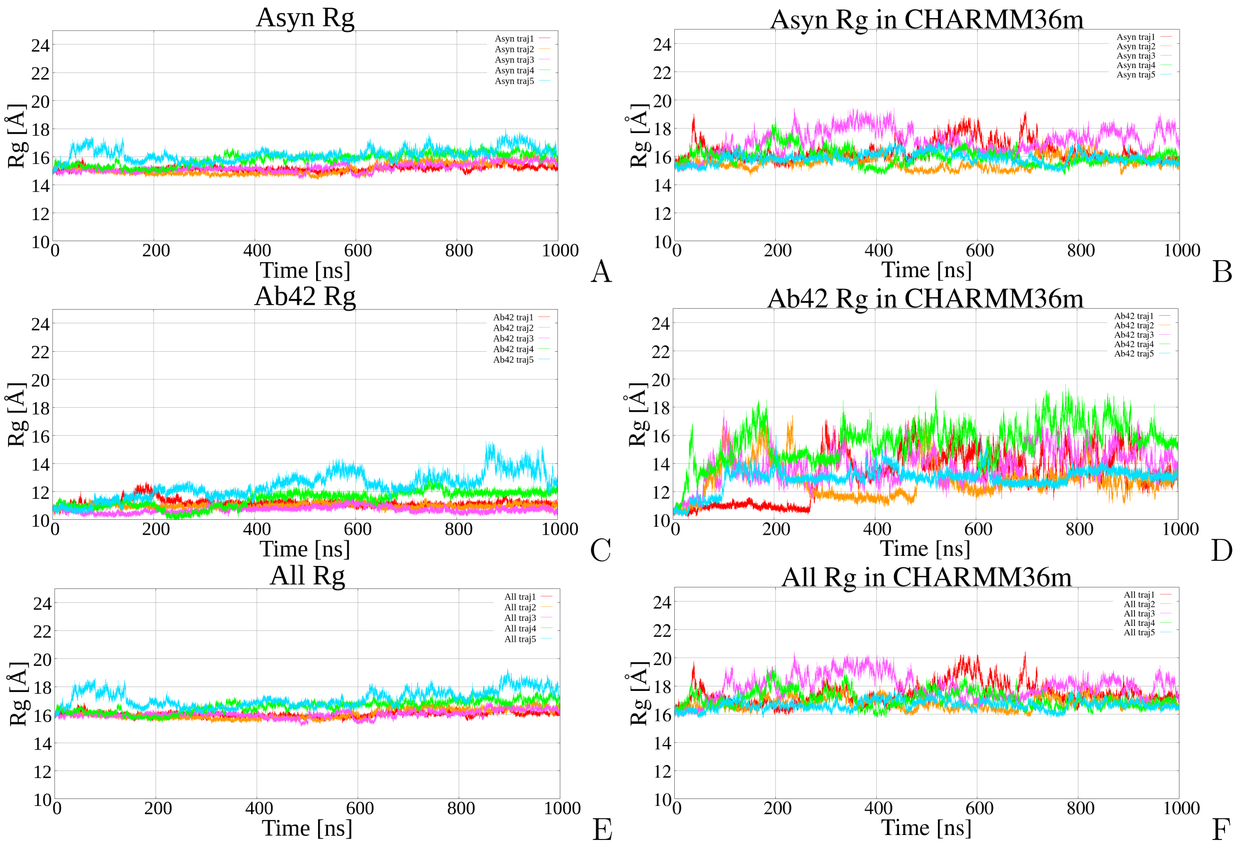

Figure S10: Plot of the Rg values for for (A-B) α-Syn, (C-D) Aβ42, and (E-F) heterodimer during explicit water simulations of the α-Syn – Aβ42 complex in AMBER-FB15 (A, C, E) and CHARMM36m (B, D, F).

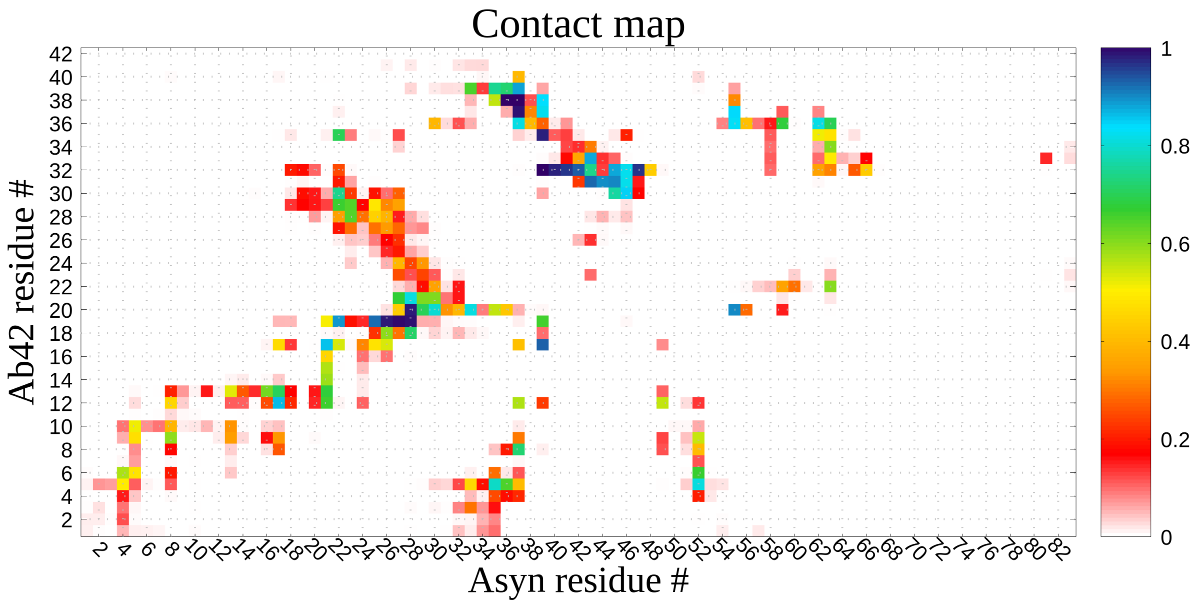

Figure S11: Contact map averaged over the last 300 ns of explicit water simulations in AMBER-FB15.

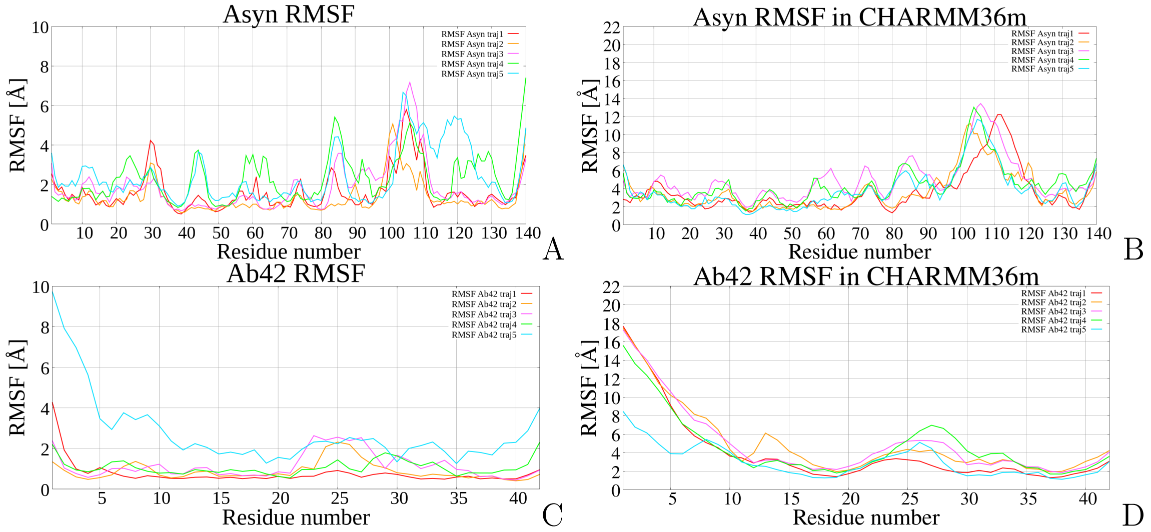

Figure S12: Plot of the RMSF values for: for (A-B) α-Syn and (C-D) Aβ42 during explicit water simulations of the α-Syn – Aβ42 complex in AMBER-FB15 (A, C) and CHARMM36m (B, D). The reference structure here is the average structure over the equilibrated part of the trajectory.

References

(1) Yang, M.; Teplow, D. B. Amyloid beta-protein monomer folding: free-energy surfaces reveal alloform-specific differences. *Journal of molecular biology* **2008**, *384*, 450–64.

(2) Zhang, Y.; Hashemi, M.; Lv, Z.; Williams, B.; Popov, K. I.; Dokholyan, N. V.; Lyubchenko, Y. L. High-speed atomic force microscopy reveals structural dynamics of α -synuclein monomers and dimers. *Journal of Chemical Physics* **2018**, *148*, 123322.

(3) Huy, P. D. Q.; Vuong, Q. V.; La Penna, G.; Faller, P.; Li, M. S. Impact of Cu(II) Binding on Structures and Dynamics of Aβ42 Monomer and Dimer: Molecular Dynamics Study. *ACS Chemical Neuroscience* **2016**, *7*, 1348–1363.

(4) Patriksson, A.; van der Spoel, D. Temperature generator for REMD-simulations. 2018 (accessed February 1, 2018).

(5) Patriksson, A.; Van Der Spoel, D. A temperature predictor for parallel tempering simulations. *Physical Chemistry Chemical Physics* **2008**, *10*, 2073–2077.
